# Multifunctional 3D-Printed Wound Dressings Containing a Combination of Synergistic Antimicrobials in the Management of MRSA Infected Topical Wounds

**DOI:** 10.1021/acsami.5c08968

**Published:** 2025-08-18

**Authors:** Iman Mattar, Guillermo Landa, Marina Frutos-Lizano, Natalia Izquierdo, Elena Tapia, Marta Perez, Lluis Lujan, Silvia Irusta, Gracia Mendoza, Manuel Arruebo

**Affiliations:** a Instituto de Nanociencia y Materiales de Aragón (INMA), CSIC-Universidad de Zaragoza, 50009 Zaragoza, Spain; b Department of Chemical and Environmental Engineering, University of Zaragoza, Campus Río Ebro-Edificio I+D, C/Poeta Mariano Esquillor S/N, 50018 Zaragoza, Spain; c Aragon Health Research Institute (IIS Aragon), 50009 Zaragoza, Spain; d Animal Unit, University of Zaragoza, 50009 Zaragoza, Spain; e Department of Anatomy, Embryology and Animal Genetics, University of Zaragoza, 177 Miguel Servet Street, 50013 Zaragoza, Spain; f Instituto Universitario de Investigación Mixto Agroalimentario de Aragón (IA2), University of Zaragoza, 50013 Zaragoza, Spain; g Department of Animal Pathology, University of Zaragoza, 177 Miguel Servet Street, 50013 Zaragoza, Spain; h Department of Pharmacology and Physiology, Forensic and Legal Medicine, Veterinary Faculty, University of Zaragoza, 177 Miguel Servet Street, 50013 Zaragoza, Spain

**Keywords:** 3D printing, wound dressings, antimicrobial therapies, wound healing, MRSA

## Abstract

Despite increased pre- and postoperative care and aseptic practices in surgical rooms, methicillin-resistant *Staphylococcus aureus* (MRSA) continues to colonize acute surgical wounds. MRSA is also present in chronic nonhealing wounds, such as diabetic foot and pressure ulcers. In this work, advanced antimicrobial-loaded wound dressings are 3D printed using fused deposition modeling. To achieve a high antimicrobial effect, the topical antiseptic octenidine (OCT) was incorporated into the pellets used in the feeder of the extruder prior to fused modeling. Lysostaphin (LYS), a lytic enzyme that cleaves MRSA peptidoglycan, was incorporated by supramolecular interactions on the surface of the OCT-loaded dressings to exploit the anti-MRSA synergy identified here between OCT and LYS showing a fractional inhibition concentration index (FICI) of 0.156. Minimum inhibitory concentration (MIC) and bactericidal concentration (MBC) values for the OCT were 1 and 25 μg/mL, respectively, whereas the MIC and MBC values for the LYS were 0.1 and 0.2 μg/mL, respectively. The resulting dressings completely eradicate MRSA USA 300 inocula (10^5^ CFU/mL) in 96 h. The bactericidal mechanisms exerted by these dressings were identified through molecular techniques, showing lytic effects on the cell wall peptidoglycans of treated bacteria. Additionally, OCT at 1 μg/mL was able to reduce lipopolysaccharide (100 ng/mL)-induced NO production on murine J774A.1 macrophages by more than 90% demonstrating its simultaneous anti-inflammatory action. This effect was also corroborated by the qRT-PCR analysis of several pro-inflammatory genes including IL-1β, IL-6, TNF-α, and Nos2. The combination of OCT and LYS within the dressings reveals higher *in vivo* therapeutic effects compared to free compounds or individual antimicrobial-loaded dressings. *In vitro* and in preclinical models, the use of OCT-LYS dressings effectively reduces MRSA bioburden and inflammation, promoting fast wound healing.

## Graphical abstract

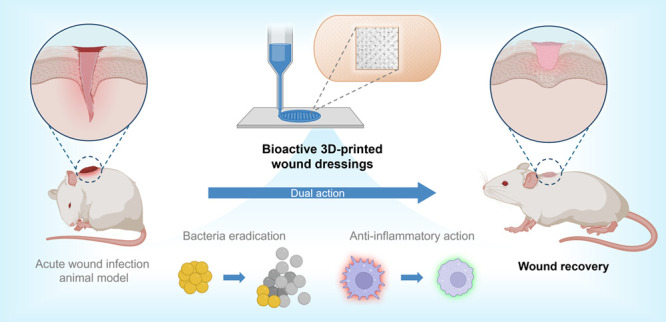

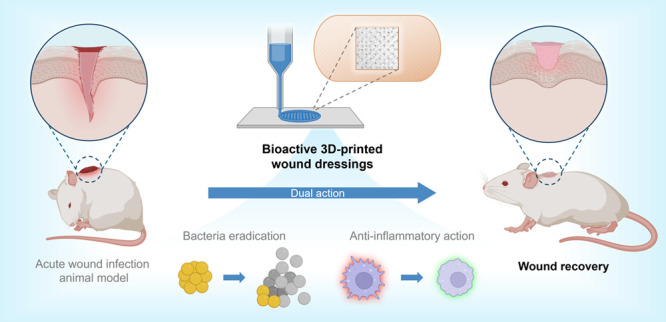

## 1 Introduction

The 2019 estimates of the global burden of antimicrobial resistance revealed that *Staphylococcus aureus* represents the second leading pathogen for deaths associated with resistance.^1^ More than 100 000 deaths worldwide were attributed in 2019 to Methicillin-resistant *Staphylococcus aureus* (MRSA), which is a strain that carries the mobile genetic element staphylococcal chromosomal cassette mec (SCCmec) encoding for specific penicillin-binding proteins (i.e., PBP2a) that confer resistance against several β-lactam antibiotics (such as methicillin).^2^ The European Antimicrobial Resistance Surveillance Network described a significant decreasing trend in the number of reported MRSA bloodstream isolates in the EU/EEA during the 2018–2022 period, but still its presence remains alarmingly high in several other European countries; in addition, numerous MRSA strains multiresistant to other different antibiotic classes (e.g., macrolides, aminoglycosides, quinolones, etc.) have been identified.^3^

*S*. *aureus* is a facultative commensal bacteria commonly present asymptomatically on the skin, nasopharynx, intestine, and mucosa of healthy individuals but due to different endogenous (e.g., weakened immune system, wounding, diabetes, etc.) or exogenous (e.g., specific medications, smoking, implants, etc.) factors, it can become opportunistic and can produce skin infections, bacteremia/sepsis, pneumonia, endocarditis, topical wound infections, and so on.^4^ Despite increased pre- and postoperative care, MRSA can colonize surgical wounds with infection rates varying depending on the region analyzed. A 2016 European study reported a 0.06% MRSA-related surgical site infection rate, while data from India (2009–2012) showed a 1.38% rate.^5,6^ In Japan (2012–2018), MRSA infections ranged from 0.03% in laminectomies to 2.33% in amputations.^7^ In the U.S. (2009–2010), MRSA infection prevalence among major surgeries was reported as 1.03%.^8^

Not only in surgical wounds but also MRSA is alarmingly present in chronic nonhealing wounds such as diabetic foot, pressure, and venous and arterial insufficiency ulcers. For instance, a meta-analysis of 112 studies, involving 16 159 diabetic foot patients, from which 22 198 microbial isolates were sampled, identified *S*. *aureus* as the most common pathogenic bacteria observed, MRSA being identified in 18% of those samples.^9^ The analysis of the chronic wound microbiome of 2963 U.S. patients identified *Staphylococcus* as the most prevalent genus, and 25.2% of the wounds, examined using 16S rDNA pyrosequencing, were colonized with MRSA.^10^ Similar data were obtained in several other meta-analyses.^11^ For many of those chronic wounds, antimicrobial treatments fail to heal. For instance, a retrospective analysis of 38 patients receiving ambulatory treatment of their MRSA-infected leg ulcers during 1 month consisting of the use of silver wound dressings and daily antiseptic treatment and hygiene measures failed in 22 of them.^12^ Even combinatorial treatments are unable to eradicate infective bacteria on chronically infected wounds. For instance, a randomized, controlled clinical study with 43 patients having 88 infected diabetic foot ulcers revealed that no statistical benefits were observed for patients receiving bioresorbable antibiotic (gentamicin)-collagen topical sponges with systemic antibiotics (levofloxacin or amoxicillin-clavulanate) over those receiving just the systemic antibiotics.^13^ In the evaluation of the effectiveness of different interventions proposed for the management of infected chronic foot ulcers in diabetic patients, Vas et al. found limited evidence for the benefits of combinations of antimicrobials and honey or the use of growth factors and cellular products or the use of topical oxygen therapy or the use of electromagnetic radiation (i.e., electrical, light, magnetic) over the best standard care.^14^ Furthermore, even after the analysis of commercially available products on the market, Weigelt et al. concluded that the number of high-quality randomized controlled trials in which scientific evidence demonstrates the effectiveness of antibiofilm compounds for chronic wound healing is scarce.^15^ Therefore, more research in that field is needed to provide the best scientific evidence to address this unmet clinical need. In response, we decided to identify a synergetic combination of antimicrobials having different mechanisms of antimicrobial action to reduce the chances to develop resistance.

After wounding, a perfectly orchestrated physiological process starts with the formation of a fibrin-based clot (homeostasis), inflammatory cell recruitment, neo-angiogenesis, epithelialization, granulation tissue formation, and remodeling. But as we mentioned before, due to endogenous or exogenous factors, wound healing can be delayed or become even chronic due to the presence of pathogenic bacteria. The pathophysiology of those difficult-to-treat wounds is commonly represented by the presence of a polymicrobial dynamic microbiome, ubiquitous mature bacterial biofilms, persistent inflammation, high levels of neutrophil-derived proteases, and in some cases intracellular *S*. *aureus* persisters (named small colony variants).^10^ Those phenotypic characteristics make bacteria refractory and resilient against the host′s defenses and against most antimicrobial treatments systemically applied and/or topical.

Wound debridement (only when revascularization is feasible) and antiseptic solutions (e.g., chlorhexidine, iodopovidone, octenidine, polyhexamethylene biguanide, hypochlorous acid, etc.) are commonly applied as pharmacological treatments on those infected chronic wounds together with the concomitant treatment of underlying causes that might delay healing and with the recommended reduction of potential risk factors.^16^ The use of topical antibiotics (e.g., mupirocin, fusidic acid, bacitracin, minocycline, gentamicin, etc.) is generally restricted for those cases in which infection is extended to the bone (osteomyelitis) to avoid the potential development of antibiotic resistance, contact dermatitis, and potential impaired wound healing.^17^

Not only antibiotics and antiseptics are prescribed but also antimicrobial peptides are used to eliminate pathogenic bacteria from chronic nonhealing wounds and concomitantly boosting the host′s immune response by regulating inflammation and promoting homeostasis.^18^ More than 500 anti-MRSA peptides have been identified in the literature.^19^ Some of those antimicrobial peptides (e.g., oritavancin, daptomycin, etc.) have been approved for their use on topical skin infections associated with Gram-positive bacteria. Some of them show intracellular targets, but most of them electrostatically bind to negatively charged lipoteichoic acids of Gram-positive bacteria.^20^ Their clear advantage compared to antibiotics is that they target multiple mechanisms of bacteria, while their advantage over antiseptics is their reduced cytotoxicity on eukaryotic cells. One of them, lysostaphin (LYS), a type IIIa bacteriocin (i.e., lytic enzyme), is able to cleave the cross-linked pentaglycine bridges in *S*. *aureus* peptidoglycan.^21^ Positive preclinical and clinical results on the management of infection have been reported, and even the human immunogenicity associated with its parenteral administration has also been successfully overcome by developing deimmunized LYS.^22^ Synergistic combinations of this lytic enzyme with other antimicrobial peptides have shown superior antimicrobial activity in a rabbit model of MRSA topical infection than either of those antimicrobials as monotherapy.^23^ The combination of LYS with antimicrobial and anti-inflammatory peptides has also demonstrated, in a murine model of intradermally MRSA infection, superior bactericidal activity and anti-inflammatory response than monotherapy.^24^ Intracellular MRSA has been successfully eliminated from infected macrophages after intraperitoneal MRSA injection in mice using the simultaneous action of LYS with vancomycin when loaded within mannosylated exosomes taking advantage of the high levels of mannose receptors in macrophages.^25^ We have previously demonstrated that the nanoencapsulation of LYS in PLGA (poly­(lactic-*co*-glycolic acid)) nanoparticles preserves its antimicrobial action against planktonic and sessile MRSA.^26^ Furthermore, it has been previously reported that LYS is unable to eliminate intracellular pathogens due to its size (∼25 kDa) and due to its activity reduction after lysosomal entrapment, but we previously demonstrated that LYS-encapsulated in PLGA is also able to eradicate intracellular MRSA, attributed to the protection provided by the polymer and due to its promoted intracellular uptake when loaded within nanoparticles.^26^ Some other peptides have demonstrated not only antimicrobial effects but also immunomodulatory action suppressing lipopolysaccharide (LPS)-induced inflammation *in vitro* and promoting re-epithelialization and angiogenesis *in vivo*.^27^

As we mentioned before, there are several antiseptics available for the management of MRSA-associated infected wounds. Octenidine (OCT), a cationic dihydropyridine, shows broad spectrum efficacy, low allergenic properties, and superior cytocompatibility compared to other antiseptics.^28–30^ Its mechanism of antimicrobial action is based on hydrophobic and electrostatic interactions with the negatively charged membrane wall causing membrane reorganization and mechanical disruption with the consequent cell lysis.^31^ Permeabilization, neutralization, and disordering of the lipid bilayer of Gram-positive bacteria have also been identified as nonlipid specific and simultaneous mechanisms of antimicrobial action.^32^ It has also been demonstrated that clinical isolates of MRSA did not acquire stable resistance after a continuous exposure to reduced concentrations of OCT.^33^ Anti-inflammatory and protease-inhibitory ability have also been reported for OCT *ex vivo* and *in vivo*.^34,35^ In a comparative preclinical study on the treatment of MRSA biofilm-infected wounds, OCT (500 ppm) impregnated gauzes outsmarted mupirocin (2%) treated wounds showing higher reduction in MRSA counts and in the number of inflammatory cells (6 times less comparing OCT treated wounds with the untreated controls and halved compared to mupirocin counts).^36^ A prospective study in 44 patients having 49 venous leg ulcers treated with commercially available advanced silver-based wound dressings alone, OCT alone or OCT and advanced silver-based wound dressings in combination revealed a superior reduction in the wound bioburden and faster granulation tissue formation when using just OCT.^37^

Herein, we demonstrate that OCT and LYS are synergetic antimicrobials that potentially reduce the chances for MRSA to develop resistance. Despite of the fact that MRSA resistance to LYS has been reported, its combination with the broad spectrum unspecific antiseptic OCT would decrease the chances for bacteria to develop phenotypic mutations or the acquisition by horizontal or vertical gene transfer due to the synergetic combination of their unspecific and multiple mechanisms of antimicrobial action.^38^ We demonstrate in a murine model of excisional topical infected wound not only a reduction in the bacterial bioburden but also a measurable therapeutic reduction in the inflammatory reaction when using OCT-LYS loaded 3D printed wound dressings.

To the best of our knowledge, this is the first time that a synergetic effect has been identified between LYS and OCT and validated *in vivo*. In addition, the anti-inflammatory effect of OCT has been demonstrated using the LPS-induced immune response on murine macrophages leading to inflammation *in vitro* and corroborated by qRT-PCR analysis of several pro-inflammatory genes including IL-1β, IL-6, TNF-α and Nos2.

## 2 Results and Discussion

We initially screened which antimicrobials could be suitable for fused deposition modeling (FDM) 3D printing. As mentioned before, due to the potential low antimicrobial loading achieved when immersing an already printed dressing in an antimicrobial solution, we decided instead to include the selected antimicrobial together with the polymer in the feed of the 3D printer before printing. By doing so, all of the printed dressings would contain in their structures a large amount of the selected antimicrobial. Due to the high temperatures reached in the printer head during the thermal extrusion (i.e., 210−220 °C), the thermal stability of the antistaphylococcal topical antiseptics chlorhexidine digluconate and OCT, together with the antibiotic fusidic acid, was evaluated. Figure 1A shows that before the thermal treatment the three antimicrobials were able to completely eliminate MRSA bacteria in its planktonic form having MBCs of 5 μg/mL for both chlorhexidine digluconate and fusidic acid and 25 μg/mL for OCT. Despite the fact that fusidic acid and chlorhexidine digluconate showed superior efficacy (i.e., lower MICs (minimum inhibitory concentrations) and MBC (minimum bactericidal concentrations) values) over OCT, we observed that after heating them (at 220 °C for 5 min, conditions reached in the printer head during printing) only, the OCT preserved its antimicrobial action (Figure 1A), while the other two antimicrobials lost their activity at the doses tested. MIC and MBC values for the heated OCT were 1 and 25 μg/mL, respectively. Those MIC and MBC results obtained for the antimicrobials before heating are in agreement with previous data.^39,40^

**Figure 1:**
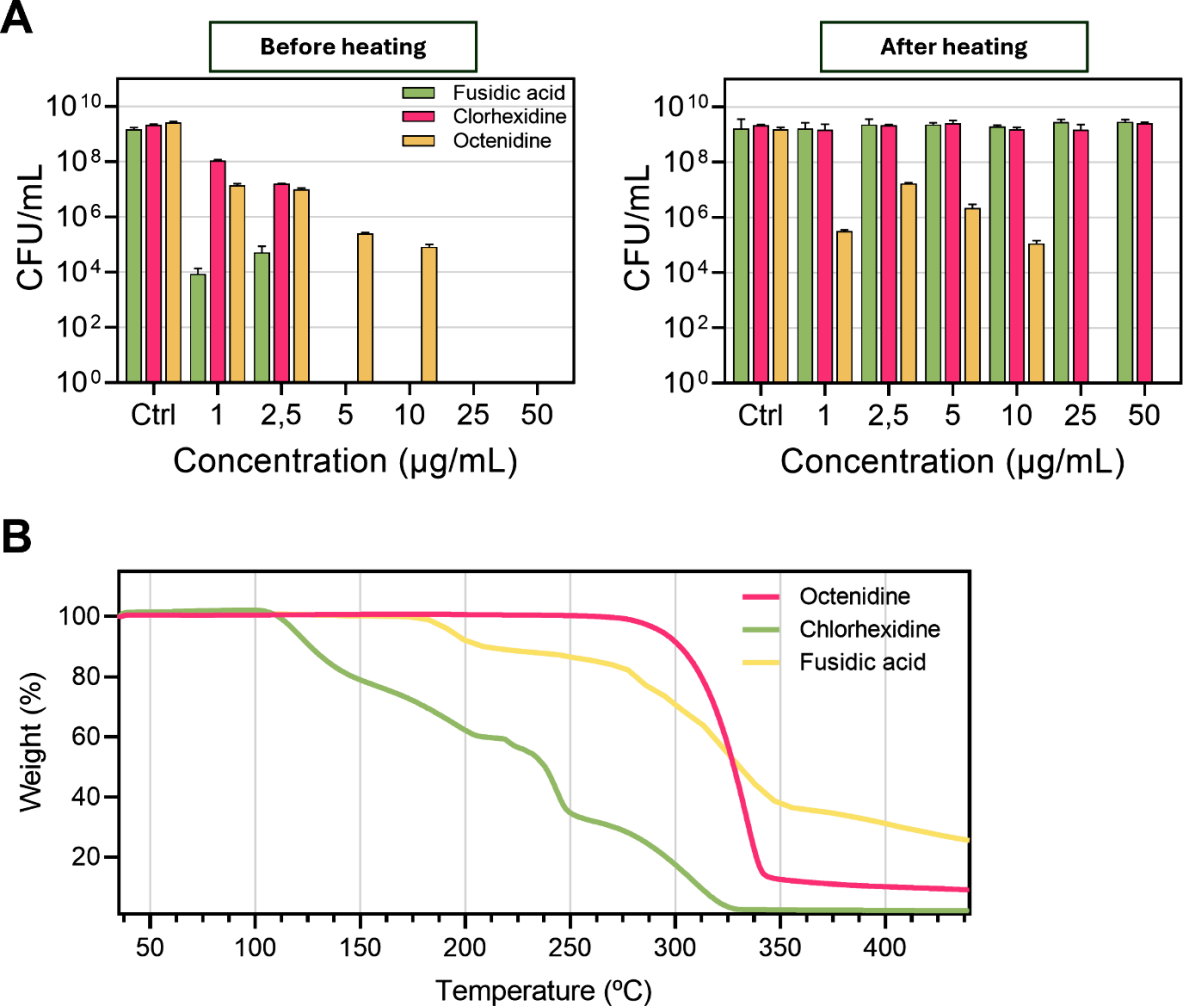
Analysis of antimicrobial activity and thermal stability of selected antimicrobials. (A) Antimicrobial activity assay against MRSA before and after heat treatment at 220 °C for 5 min. Results are presented as the mean ± standard deviation (SD) from three independent experiments (*n* = 3). (B) TGA (thermogravimetric analysis) thermograms of the antimicrobials in air.

These previous results are also in agreement with the subsequent TGA results retrieved for the selected compounds. Figure 1B shows that no measurable weight loss was observed for the OCT until reaching temperatures as high as 273 °C, while fusidic acid and chlorhexidine showed important weight losses starting at 194 and 182 °C, respectively.

Previous studies are also aligned with our results, showing that chlorhexidine digluconate undergoes exothermic decomposition starting at ∼160 °C, while fusidic acid starts to partially decompose at 155 °C.^41,42^ On the other hand, the melting point of OCT was reported to be located at temperatures above 214 °C.^43^ Above this temperature, the weight loss can be attributed to the loss of the dihydrochloride groups, which was confirmed by the change in the hydrophilic nature of the resulting heated antiseptic as we mentioned before. On the contrary to the parent OCT dihydrochloride, we observed that the heated OCT was sparingly soluble in water. Also, both the water angle measurements for the OCT-loaded dressings and the OCT release kinetics described below confirmed that observation.

Many commercially available adhesive bandages have a polymeric porous top layer placed on the cotton-based pad to prevent them from sticking to the wound. Therefore, we decided to fabricate 3D printed mesh-type self-supported dressings to demonstrate the versatility of the FDM 3D printing technique, which is potentially applicable in commercial wound dressings. Herein, FDM 3D printing was selected as an additive manufacturing technology to print antimicrobially loaded layers due to its cost efficiency, speed, and polymer versatility. Despite the use of high temperatures during the printing process and the lower resolution achievable by 3D printing compared to other material processing techniques (e.g., electrospinning), the low cost of this technology, the straightforward large scale fabrication and rapid prototyping achievable, and the easy fabrication of complex geometries in the macroscale represent clear advantages of this additive technology. Figure 2A shows that OCT-loaded polymeric (PLA and PLA:PEO (polyethylene oxide blends) mesh-type dressings can be 3D printed starting from polymer pellets with or without containing OCT. A clear color change from transparent to brownish was also indicative of the presence of OCT in the loaded dressings. Those two thermoplastic polymers were chosen for their cost-effectiveness, biodegradability, compostability, and easy processability by 3D printing. The porous mesh-type structure was chosen to allow gas permeation and exchange and to avoid potential wound maceration while maintaining an adequate moisture on the wound bed and facilitate subsequent regenerative cells migration during wound healing.

**Figure 2:**
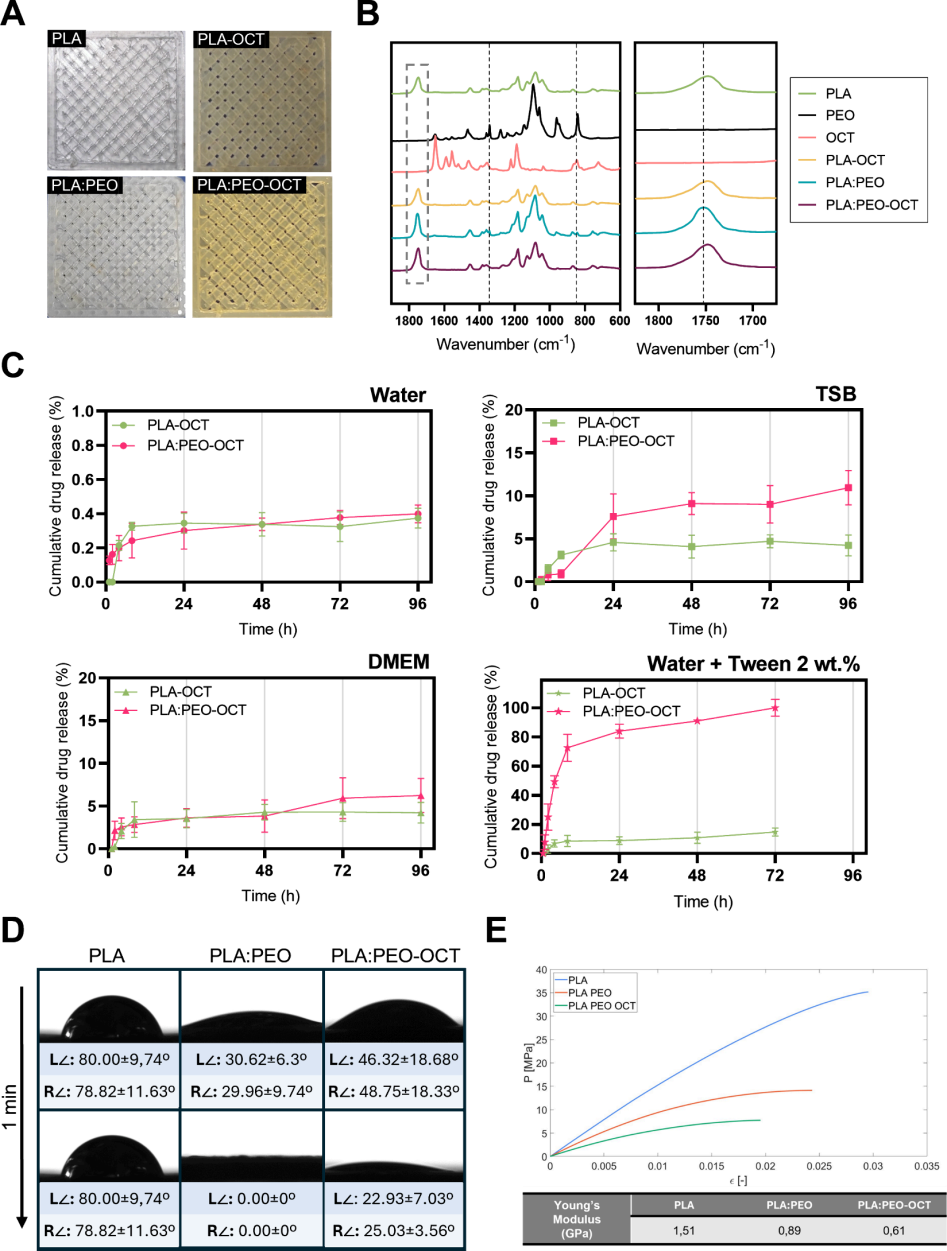
(A) Images of 3D printed dressings. (B) FTIR (Fourier transform infrared spectroscopy) analysis of printed dressings and those of the free antimicrobial compounds. (C) OCT release profile in different media under sink conditions; data are presented as the mean ± SD (*n* = 5). (D) Contact angle data and images of water droplets on the printed materials over time (1 min). (E) Mechanical testing stress–strain graph on the 3D printed materials and their corresponding Young’s moduli.

PEO, as mentioned before, was incorporated into the blend to increase the hydrophilic nature of the final dressings and, consequently, to favor wound exudates absorption. Figure 2B shows FTIR spectra of PLA, PEO, and OCT and their combinations. PLA:PEO sample spectrum showed the characteristic PLA bands. The CH_3_ stretching band was observed at 1454 cm^–1^, and signals related to the ester group (1277 cm^–1^), the νO–C asymmetric mode (1080 cm^–1^), and the –OH bending (1044 cm^–1^) could also be observed, in agreement with the previous literature.^44^ The band at 1210 cm^–1^ appeared only as a shoulder and no band was observed in the wavenumber around 920 cm^–1^, while an important peak at 1180 cm^–1^ and a signal at 956 cm^–1^ were detected. This would indicate that the PLA loses its semicrystalline nature after printing becoming amorphous as previously reported.^45,46^ Besides, the presence of PEO was confirmed by bands at 1342 cm^–1^ assigned to wagging vibrations and at 844 cm^–1^ related to asymmetric rocking vibrations both of CH_2_ groups.^47^ It is interesting to notice that the shift of the C=O bond stretching of PLA from 1748 to 1752 cm^–1^ due to the presence of PEO might suggest a supramolecular interaction between both polymers through this group as previously reported.^48^ On the other hand, the addition of OCT seemed to affect that interaction since, in the spectrum of the sample with the three compounds, the C=O band appeared again at 1748 cm^–1^ (Figure 2B inset). Other effect of the addition of the OCT was the disappearance of the 1342 and 844 cm^–1^ vibrations, attributed to the presence of PEO.

Figure 2C shows the different OCT release profiles in different media under sink conditions. Almost no release (<1 wt %) was detected in water (circles) due to the previously mentioned hydrophobic nature of the thermally printed OCT, whereas in bacterial cell culture medium (TSB, trypto-casein soy broth) (squares) after 96 h more OCT (∼11 wt %) was released when loaded in PLA:PEO than when loaded in PLA (∼4 wt %). Probably, the hydrophilic PEO favors the transport and diffusion of OCT to the release medium. The same behavior was observed in the eukaryotic cell culture medium (DMEM, Dulbecco’s modified Eagle medium) (triangles) (released amounts of ∼6.5 and ∼4 wt %, were measured from PLA:PEO-OCT and PLA-OCT based dressings, respectively). The polypeptide and amino acids content of both cell culture media (casein and soy peptone in TSB and glutamine in DMEM) acting as mild surfactants could be responsible for facilitating the diffusion and release of the hydrophobic OCT from the 3D printed dressings from both polymers. We corroborated those findings by using a strong surfactant, Tween-20 (a polysorbate-type nonionic surfactant) at 2 wt % in water, and we observed after 72 h a superior OCT release (∼98 wt %) (stars) from the PLA-PEO-OCT dressings compared to that retrieved from the PLA-OCT printed samples (∼10 wt %) and a superior OCT release than in any of the other release media tested. Tween-20 forms emulsions in water at concentrations as low as 0.06 wt % (i.e., critical micellar concentration); therefore, hydrophobic OCT could be easily entrapped within Tween-based micelles and facilitate its diffusion to the polar medium.

Figure 2D shows the water contact angle on the printed dressings during 1 min in contact on the sample stage using an optical tensiometer. PLA:PEO blends showed a superior hydrophilic character over PLA printed mesh-type dressings, and the incorporation of OCT in the final dressings did not significantly change their wettability and superior hydrophilicity. PEO, due to its hydrophilic nature, absorbs water, forming a swollen hydration layer and becoming a hydrogel, where water is linked by hydrogen bonding to the polymer backbone.^49^ This ability is beneficial because one of the requirements of a wound dressing is having the ability to absorb wound exudates, relieve tissue edema, and avoid fluid leakage and potential bacterial dissemination while preventing maceration of the wound bed and skin edges. Thus, the hydrophilic nature of PEO would also help prevent dressing adhesion to the wound and the potential trauma upon removal. Figure 2E shows the strain–stress graph for the 3D printed dressings. Young moduli of 1.51, 0.89, and 0.61 GPa were obtained from the slope in the linear elastic region of the materials for the PLA, PLA:PEO, and the OCT-loaded PLA:PEO printed dressings, respectively. The measured tensile Young modulus of printed PLA was lower than that of the pristine one (3.5 GPa) probably because of the porosity of the printed mesh type dressing, its thermal transition from semicrystalline to amorphous (according to our FTIR results), and the reduction in the polymer MW after printing as previously reported.^50^ The stiffness of PLA was reduced by the addition of PEO as expected. The further reduction in the mechanical strength of the PLA:PEO-OCT dressings can be attributed to the load transfer to the amorphous OCT present in the amorphous polymeric blend. This reduction in the mechanical stiffness would be beneficial to allow elastic deformation and make the resulting dressings conformable to the wound bed.

Figure 3A shows the antimicrobial effect of PLA-OCT and PLA:PEO-OCT dressings over time (concentrations presented as total dressing weight per volume adjusted to contain all of the same OCT content). As it is shown, both neat polymers did not show any antimicrobial effect but when OCT was incorporated in the printed dressings, a strong antimicrobial action was observed. Using the same inoculum and analyzing the bacterial counts over time, we observed that the PLA-OCT dressings were able to reduce the bacterial burden by 3 log. However, the PLA:PEO-OCT dressings were able to completely eliminate the total bacterial load in 96 h. This observation was attributed to the high water uptake of PEO forming a swollen hydration layer which would facilitate OCT release and a promoted bacterial contact. It has been reported that *S*. *aureus* bacteria are strongly bound to hydrophilic abiotic surfaces using cell wall proteins while those cell wall proteins are weakly bound when those bacteria are tethered to hydrophobic surfaces.^51^ A superior bacterial growth on the PLA:PEO dressings (lacking in OCT) over PLA dressings (lacking in OCT) would also corroborate this observation. A sustained OCT release could be expected from the highly hydrophobic PLA-OCT dressings, whereas a fast and continuous OCT release was observed for the PLA:PEO-OCT dressings. Probably all the OCT entrapped within the PEO structure diffuses rapidly to the culture medium thanks to the ability of the polymer to absorb water and dissolve. The proximity of the bacteria to the hydrophilic PLA:PEO-OCT dressings would also contribute to the superior antimicrobial effect observed.

**Figure 3:**
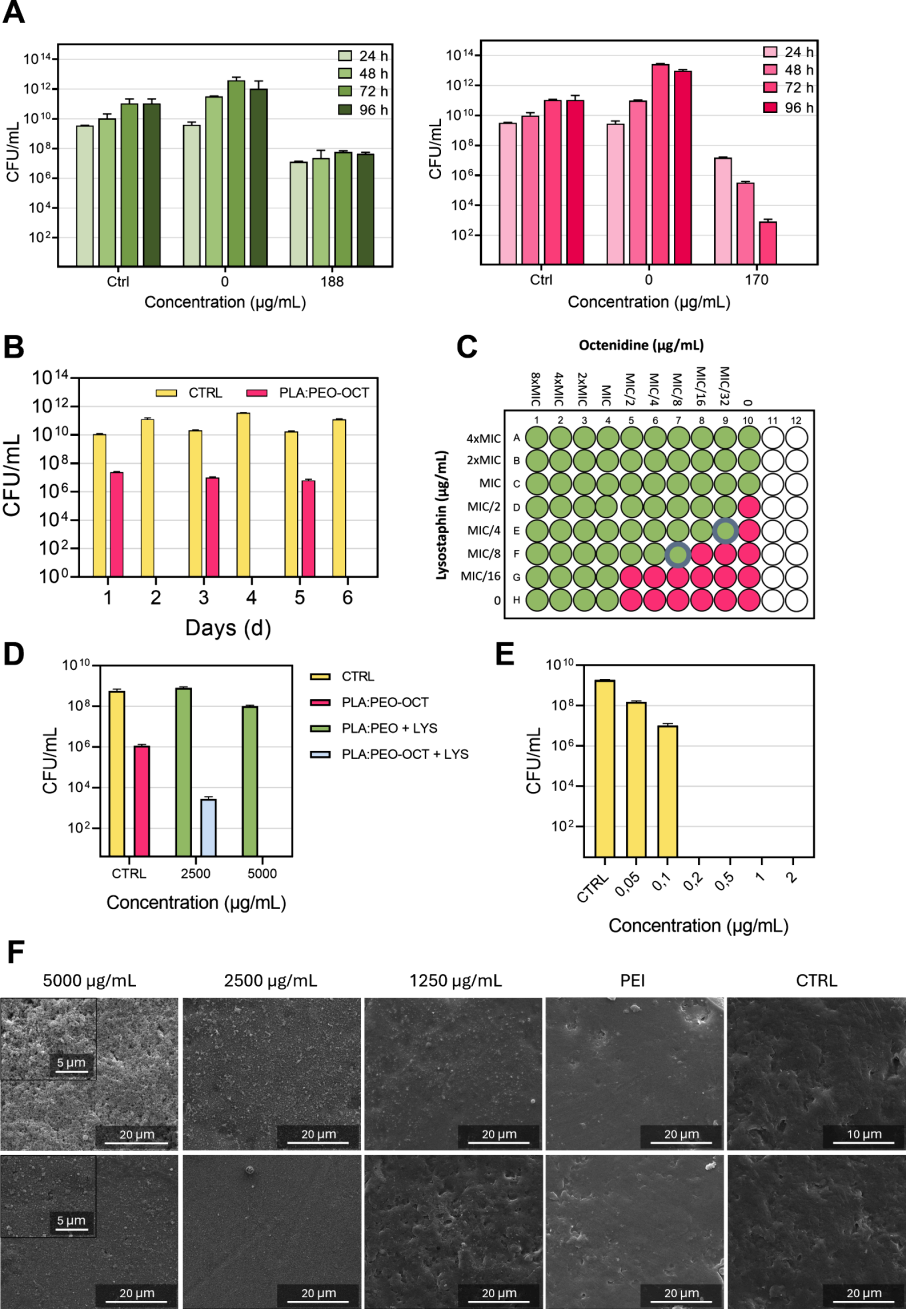
Evaluation of antimicrobial activity and synergism of PLA and PLA:PEO-based dressings against MRSA. (A) Antimicrobial activity of PLA-OCT (left) and PLA:PEO-OCT (right) printed dressings (concentrations presented as total dressing weight per volume adjusted to contain all the same OCT content). (B) Durability test of the PLA:PEO-OCT dressings. A fresh MRSA inoculum was added at days 1, 3, and 5 on the same single dressing. (C) Fractional inhibitory concentration index (FICI) analysis to show the synergism identified between the OCT and LYS against MRSA. (D) Antimicrobial assay of 8 mm in diameter dressings coated with different LYS concentrations (concentrations presented as PLGA nanoparticle suspensions at 5000 and 2500 μg/mL (which corresponds with a LYS loading of 0.8 ppm and 0.4 ppm, respectively). (E) Evaluation of the LYS MBC and MIC against MRSA. (F) SEM images of dressings with particles. SEM images, first row: PLA:PEO dressings were immersed in various LYS-loaded PLGA particle concentrations (from 1250 to 5000 μg/mL). Second row: PLA:PEO-OCT dressings immersed in various LYS-loaded PLGA particle concentrations (from 1250 to 5000 μg/mL). Bar graphs show the mean ± SD (*n* = 3 independent experiments).

We also performed a repeated bacterial challenge by exposing the same PLA:PEO-OCT dressing to successive MRSA loads (3 inoculums) every 2 days to evaluate the long-term durability of one single dressing. The results (Figure 3B) showed that in the first 24 h the dressing was able to reduce the initial bacterial burden by 3 log, and after 48 h no bacteria remained alive. We repeated the inoculation on the same used dressing up to 3 times and the results revealed large durability for the OCT-loaded dressings. Therefore, the PLA:PEO-OCT dressings were able to eliminate repeated bacterial infective loads in the time frame studied. This long-term ability is attributed to the large OCT loading present in the dressings (3.83 mg/dressing having 8 mm in diameter) thanks to the simultaneous incorporation of the antimicrobial and the polymer during the printing process in the feeder extruder.

A schematic diagram of the obtained FICI results is depicted in Figure 3C. The obtained FIC for OCT was 0.031 μg/mL (eq 2) and the one calculated for LYS was 0.125 μg/mL (eq 3) rendering a FICI (eq 1) of 0.156 which demonstrated a synergetic effect (i.e., FICI < 0.5) between both antimicrobials against MRSA. As we mentioned before, synergistic combinations for LYS have been reported when combined with antimicrobial peptides and with antibiotics.^52,53^ But to the best of our knowledge, this is the first time that synergetic effects have been reported for LYS combined with OCT. This synergetic effect could be attributed to the multiple mechanisms of antimicrobial action of both compounds combined. LYS cleaves the cross-linked pentaglycine bridges in peptidoglycan, producing the lysis of the bacteria via osmotic stress and OCT hydrocarbon chains interact with the fatty acyl chains of the bacterial membrane.^21,32,52^ This permeabilization, neutralization, and disordering of the lipid bilayer in Gram-positive bacteria have also been identified as nonlipid specific and simultaneous mechanisms of antimicrobial action for OCT.^32^ In addition, as mentioned before, low level exposure of MRSA to OCT does not induce resistance.^33^ Despite the extended use of OCT in patients, susceptibility to it in MRSA does not seem to decrease.^54^

The synergy was also observed in the antimicrobial tests performed with 8 mm in diameter PLA:PEO-OCT 3D-printed dressings, which were subsequently used in the *in vivo* study. Figure 3D shows a 3 log reduction in the MRSA cell counts when using PLA:PEO-OCT dressings. When PLGA-LYS was incorporated in the dressings, we observed a dose-dependent cytotoxic effect on bacteria attributed to the synergistic LYS effect, reaching complete bacterial eradication when using PLGA-LYS nanoparticle suspensions at 5000 μg/mL (which corresponds with a LYS loading of 0.8 μg/mL). Free LYS exhibited MIC and MBC values of 0.1 and 0.2 μg/mL, respectively (Figure 3E). When LYS was encapsulated in PLGA nanoparticles, the MIC and MBC values varied to 1.73 and 2.3 μg/mL, respectively, as reported in our previous study.^26^ Figure 3F shows the morphological analysis of the surface of the developed dressings at two different magnifications using SEM. The bare dressings show a homogeneous surface; the incorporation of PEI did not change their morphology. No phase segregation was observed between both polymers. The electrostatic assembly of the negatively charged PLGA-LYS nanoparticles on the surface of the PEI-modified PLA:PEO dressings is also shown. After repeated washings, the nanoparticles remained strongly bound to the surfaces as a result of a robust supramolecular interaction. At neutral pH the zeta potential of the developed PLGA-LYS nanoparticles is −37.7 ± 2.3 mV whereas the one of PLA layers (PEO is nonionic) is also reported to be highly negative (i.e., ∼−25 mV).^26,55^ PEI, having a strong positive electrokinetic potential at neutral pH (∼+55 mV) would favor a strong electrostatic interaction between the printed layers and the nanoparticles.^56^ A figure summarizing the physicochemical properties of the PLGA-LYS nanoparticles is included in Figure S1.

In order to analyze the mechanisms of antimicrobial action, we hypothesized that both antimicrobials would interact with the top peptidoglycan layer and reach, through the periplasmatic space, the bacterial membrane, promoting cell lysis and nucleic acids release. We consequently examined the potential bacterial membrane depolarization under the presence of OCT and LYS as shown in Figure 4A. At short times (4 h), no significant differences were observed for the treated bacteria at MICs compared to those for the nontreated controls. At longer times (24 h), a larger number of polarized cells were observed for samples treated with OCT and LYS. Therefore, both antimicrobials changed the membrane fluidity and the membrane polarization values. Therefore, membrane polarization, cell lysis, and DNA release were bactericidal mechanisms identified for the combination of both antimicrobials. Next, it was evaluated whether cell lysis induced by OCT and LYS led to the release and degradation of nucleic acids or if they were leaked intact. Even though bacteria do not have ssDNA, the evaluation of the ability of the OCT and LYS to fragment dsDNA and release ssDNA to the medium was carried out. Figure 4B shows the degradation of ssDNA over time after treatment. Compared with untreated controls, differences were observed in the relative ssDNA degradation profiles at three different time points. On the other hand, agarose gel electrophoresis using a DNA ladder (Figure 4C) revealed the circular supercoiled DNA released upon treatment. The bacterial DNA bands appeared below the last band of the molecular marker (100 bp). This is not because the DNA used had fewer than 100 base pairs but because the bacterial DNA is present as an intact genome, which is supercoiled. This supercoiling allows the DNA to migrate faster in the agarose gel due to its compact structure. The release of nonfragmented DNA was also observed in the controls, suggesting that this phenomenon occurs naturally, not solely as a result of the treatment.

**Figure 4:**
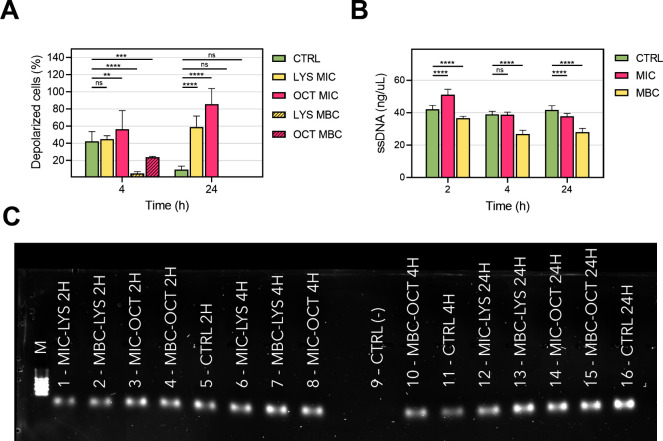
Evaluation of MRSA membrane depolarization and DNA degradation. (A) Cell depolarization assay. (B) Qubit assay for the evaluation of DNA degradation (ssDNA). (C) Agarose gel analysis of released DNA. Statistical significance between groups is indicated by horizontal bars, with *p*-values as follows: ns (nonsignificant), *p* < 0.05 (*), *p* < 0.01 (**), *p* < 0.001 (***), *p* < 0.0001 (****). Bar graphs show the mean ± SD (*n* = 3 independent experiments).

The cytotoxicity of free OCT and free LYS on human keratinocytes, fibroblasts, and macrophages is shown in Figure 5A. For the OCT a reduction in the cellular viability below 70% was reached at 5, 10, and 100 μg/mL for macrophages, fibroblasts and keratinocytes, respectively. The viability data for OCT are consistent with previously reported studies.^28,57^ The cytotoxicity assays of LYS on the same cell lines revealed that it was not cytotoxic at any concentration tested in any of the cell lines.

**Figure 5:**
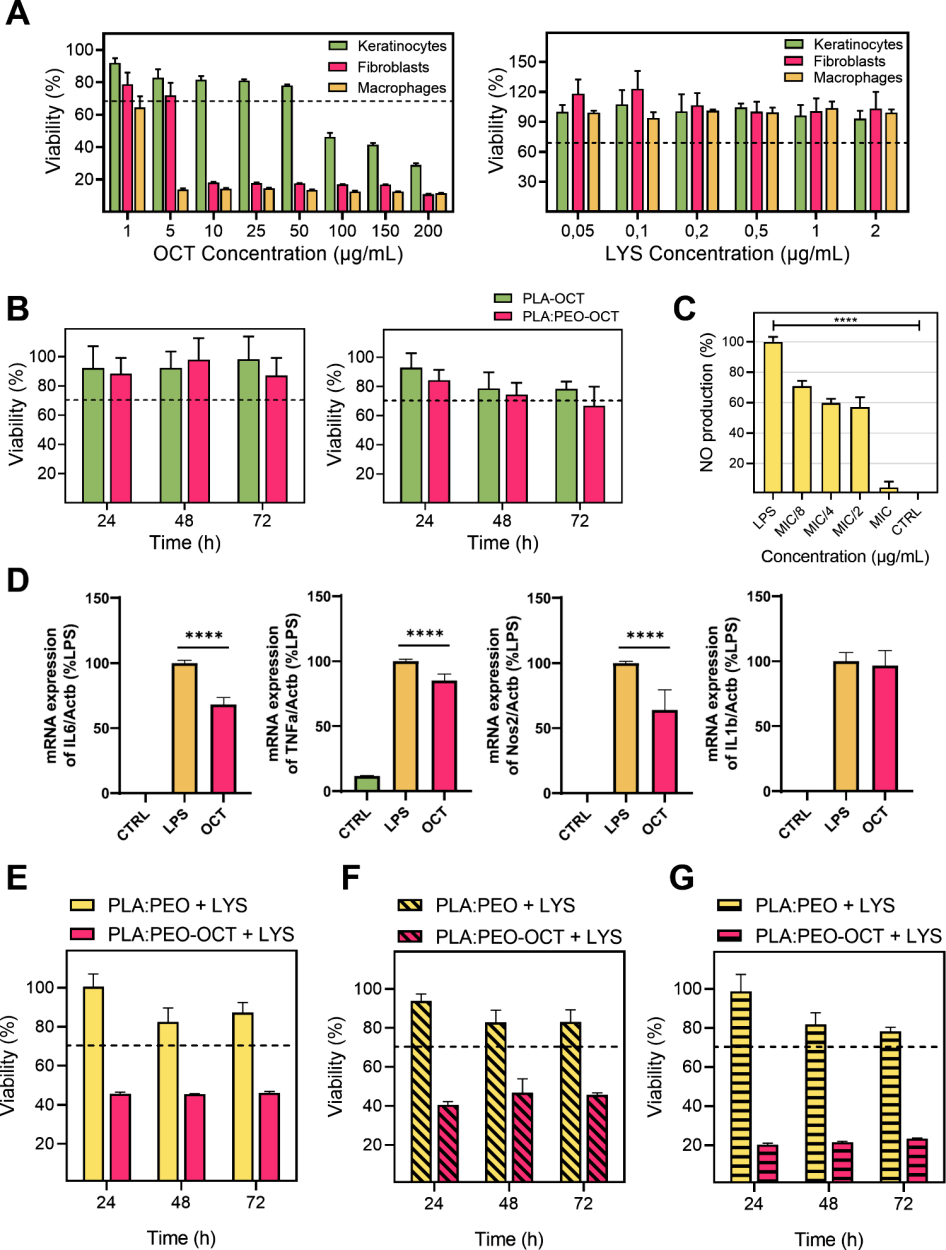
Cell viability and inflammatory response in various cell types exposed to treatments. (A) Cell viability in keratinocytes, fibroblasts, and macrophages exposed to the OCT (left) and LYS (right). (B) Cell viability in fibroblasts (left) and keratinocytes (right) when exposed to PLA-OCT and PLA:PEO-OCT dressings exudates released after 24 h. (C) Nitric oxide (NO) production after inflammatory response of LPS-induced macrophages to OCT. Statistical significance between control and all treated groups is indicated by a horizontal bar, with the *p*-value as follows: *p* < 0.0001 (****). (D) qPCR quantification of pro-inflammatory genes expression after OCT treatment (*p* < 0.0001 (****)). (E) Cell viability in fibroblasts exposed to PLA:PEO + 0.8 ppm LYS and PLA:PEO-OCT + LYS dressings (25 and 0.8 ppm, respectively). (F) Cell viability in keratinocytes exposed to PLA:PEO + 0.8 ppm LYS and PLA:PEO-OCT + LYS dressings (25 ppm and 0.8 ppm, respectively). (G) Cell viability in macrophages exposed to PLA:PEO + LYS 0.8 ppm and PLA:PEO-OCT + LYS dressings (25 ppm and 0.8 ppm, respectively). Bar graphs show mean ± SD (*n* = 3 independent experiments).

However, the exudates released after 24 h in high glucose DMEM by the corresponding OCT-loaded or LYS-loaded dressings did not show any cytotoxic effect on the same cell lines (Figure 5B,E–G). This is indicative of a strong antimicrobial action but a lack of cytotoxic effects. The slow OCT release ability of the dressings in polar solvents lacking strong surfactants (Figure 2C) might be responsible for this effect, and the low LYS loading and its reduced cytotoxic nature against eukaryotic cells can also explain its reduced cytotoxicity. However, in combination we observed a reduction in the cellular viability on fibroblasts, keratinocytes, and macrophages. The cytotoxic synergy observed against bacteria was also present against human cells. Macrophages were more sensible to the treatment probably due to their phagocytic nature, the amounts of internalized OCT and LYS probably being superior to those observed in fibroblasts and keratinocytes. It is important to point out that when cleaning an infected topical wound, antiseptics are used despite their cytotoxicity against human cells because the goal is to remove all pathogenic bacteria regardless of scarifying some eukaryotic cells because new stem cells are going to migrate and regenerate the wounded area. Therefore, the developed dressings loaded with a single antimicrobial (OCT or LYS) showed an antibiotic-like behavior in which a MRSA bacterial burden can be eliminated while large eukaryotic cell viability is preserved. In combination, cytotoxicity was observed for the combination of OCT and LYS, but as later shown in the *in vivo* results, no detrimental effects were observed after treatment with the combined dressings.

The OCT anti-inflammatory effect on macrophages is demonstrated in Figure 5C where the MIC for MRSA (subcytotoxic concentration) is effective to reduce the inflammation caused by LPS used as a common protocol to evaluate anti-inflammatory compounds, showing a nitric oxide (NO) production similar to the one for the control.^16^ In order to corroborate those findings, quantitative PCR (qPCR) of genes involved in the pro-inflammatory process induced by LPS treatment in macrophages was performed. The expression of IL-1β, IL-6, TNF-α, and Nos2 genes was evaluated, given that their involvement in the inflammatory process is widely reported in the literature. The expression of IL-6, TNF-α, and Nos2 genes showed a significant decrease in the gene expression after treatment with the OCT, with a reduction of almost 40% in the case of IL-6 and Nos2. However, OCT did not produce any reduction in the level of IL-1β expression. These results indicate that OCT principally acts on the AP-1 pathway through the mitogen-activated protein kinases (MAPKs) pathway activated by LPS.^58^ Signaling of the AP-1 transcription factor responsible for the expression of IL-6, TNF-α, and Nos2 genes is mediated through the extracellular signal-regulated kinase (ERK) and, in turn, through MAPKs signaling.^59^ Therefore, the OCT would be acting on some of the enzymes responsible for the signaling pathway. In addition, the Nos2 gene is one of the main effectors of NO synthesis;^60^ consequently, we can correlate the reduction in Nos2 expression with the observed decrease in NO production. So a multifunctional ability for OCT is here demonstrated, being able not only to eliminate pathogenic bacteria but also to simultaneously reduce cellular inflammation. This represents an outstanding advantage of our advanced dressings.

To evaluate the therapeutic potential of the proposed treatments, an *in vivo* study was conducted in a murine model of infected topical wounds, as depicted in Figure 6A. The *in vivo* study showed semiquantitative bacterial counts on the wound exudates collected with swabs from the animals, revealing a superior therapeutic effect of PLA:PEO dressings containing OCT and OCT + LYS compared to all other groups tested, as shown in Figure 6C. However, those *in vivo* results suggested that LYS did not significantly enhance the therapeutic efficacy of OCT, either in its free form or when encapsulated within the PLGA particles decorating the printed dressings. This is probably a result of the physiological reported degradation of LYS in its free form which has been overcome by its PEGylation, hydrogel encapsulation but not in this work, by PLGA-encapsulation.^61^ Probably, in this case, when released from the PLGA nanoparticles to the physiological medium, the protection was lost.

**Figure 6:**
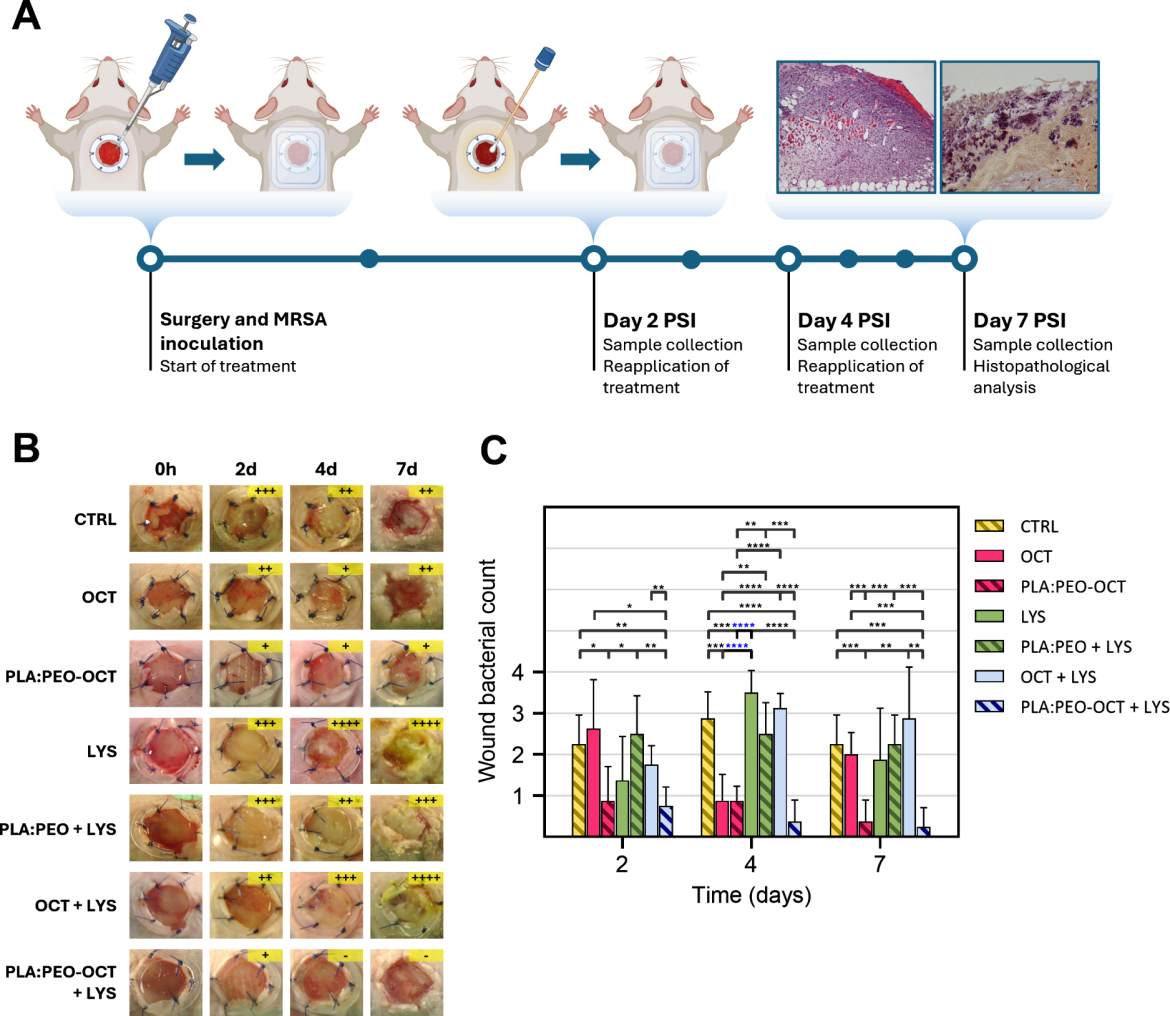
Semiquantitative bacterial counts and analysis from infected wounds in treated animals. (A) Diagram of the experimental procedure followed in the wound infection model used. (B) Images showing the progression of wounds in the different experimental groups. Bacterial growth was classified semiquantitatively using the streak plate method as follows: (−) no growth, (+) minor, (++) moderate, (+++) extensive, and (++++) massive growth. (C) Summary and analysis of the bacterial load obtained by the streak plate method. Data are presented as the mean ± SD (*n* = 8). Statistical significance between groups is indicated by horizontal bars, with *p*-values as follows: *p* < 0.05 (*), *p* < 0.01 (**), *p* < 0.001 (***), *p* < 0.0001 (****).

As it was mentioned before, MRSA can easily colonize biotic (skin, mucosa, nasopharynx, intestine, etc.) and abiotic surfaces (prothesis, indwelling medical devices, implants, cardiac pacemakers, etc.).^62^ The bacterial colonization of a surface and the consequent biofilm formation provide an evolutive advantage against the host′s immune system and against antimicrobial regimens. Also, bacteria transmit genetically to their descendants the characteristics of the surface they are attached to in order to be even faster in the adaptive adhesion to the same surface.^63^ According to the *in vivo* results, we can conclude that the combination of free OCT and LYS, which was effective in the *in vitro* assay, did not maintain its efficacy in the *in vivo* study. Additionally, LYS was ineffective in both its free and encapsulated forms, while the encapsulated form of OCT demonstrated a better effectiveness than its free form at equivalent doses. The highest reduction in the bacterial counts was observed for the combined PLA:PEO-OCT + LYS dressings at all times sampled. At day 7 PSI only one of the four sampled wounds showed a reduced number of bacterial colonies. The high experimental challenge to which the wounds were subjected (≈10^4^ CFU/mL of MRSA) was completely eliminated in three of the four wounds analyzed. The characteristic golden color of *S*. *aureus* (i.e., due to the production of a metabolic byproduct called staphyloxanthin) was present in all tested groups except in the free OCT, PLGA:PEO-OCT, and PLGA:PEO-OCT + LYS groups. This pigment helps *S*. *aureus* thrive in various conditions, and it is shown how our advanced dressings rendered the best wound care.

Histopathological evaluations were conducted to assess the wound status with respect to tissue morphology, bacterial presence, inflammation, and cellular infiltration across the experimental groups (Figure 7). The presence of bacteria in the tissues (i.e., epidermis, dermis, and subcutaneous tissue) as well as hyperplasia, ulceration, crust, inflammation, the identification of the cell types (neutrophils, fibroblasts, macrophages, and lymphocytes), fibroplasia, panniculitis, mononuclear inflammation, and necrosis are described in Table S1. Notably, the untreated groups and those treated with free LYS exhibited a greater intensity of inflammatory reaction and a larger number of bacterial colonies within the dermis and subcutaneous tissues compared to those treated with PLA:PEO + LYS and PLA:PEO-OCT + LYS, where bacterial loads and inflammatory reaction were significantly reduced. Similarly, while fibroplasia and the presence of inflammatory cells (mainly fibroblasts and neutrophils) were evident in most groups, the groups treated with PLA:PEO-OCT and PLA:PEO-OCT + LYS dressings demonstrated reduced necrosis and a circumscribed inflammatory reaction, suggesting better tissue preservation and wound healing.

**Figure 7:**
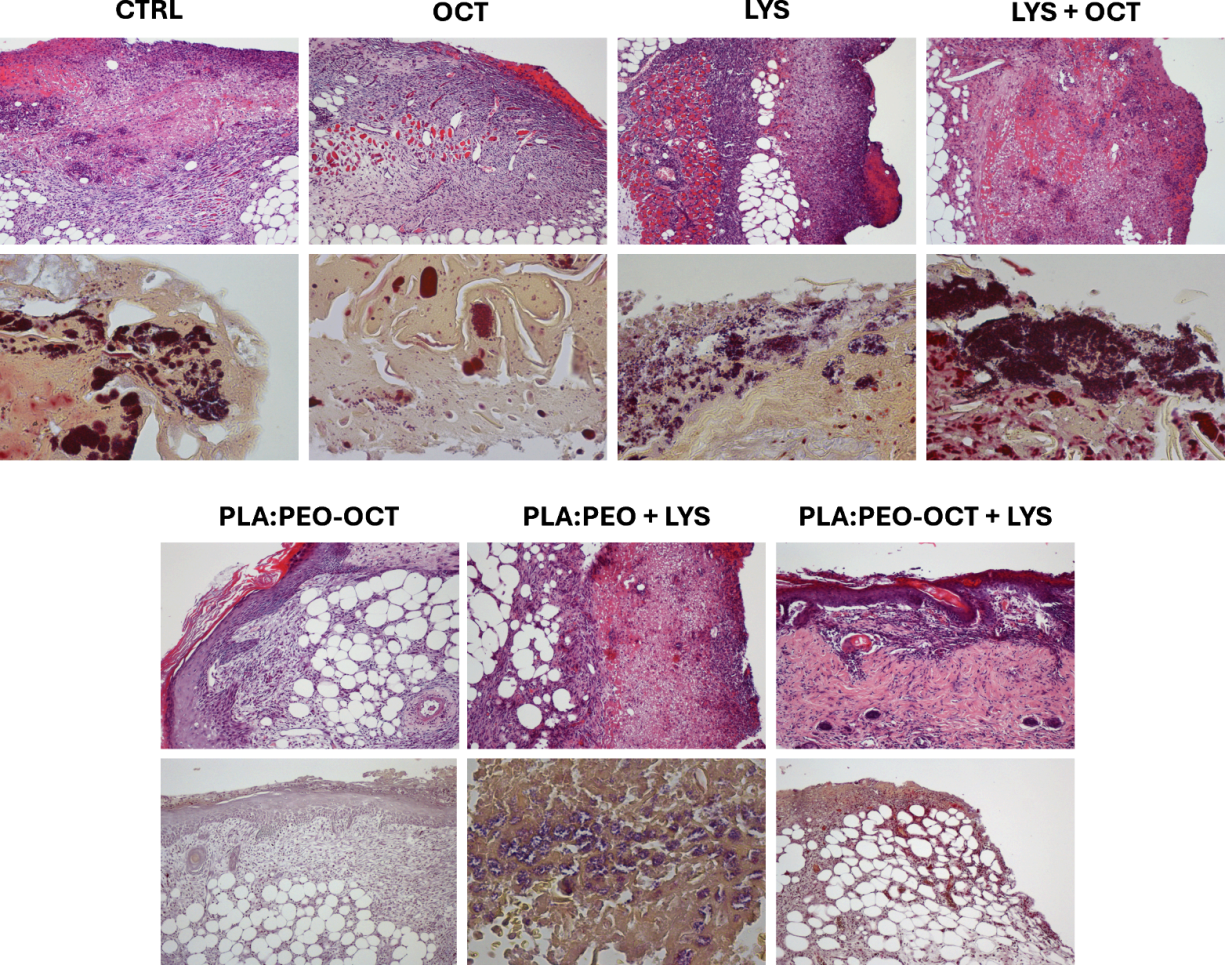
Histopathological evaluation of the experimental groups at 7 days after surgical intervention (PSI). Upper row: hematoxylin–eosin staining (all images taken at ×4 magnification). Lower row: Gram staining (mostly at ×60 magnification except for PLA:PEO-OCT and PLA:PEO-OCT + LYS, which were taken at ×10 magnification).

In terms of structural recovery, epidermal thickening and ulceration were effectively controlled in the groups receiving the PLA:PEO-OCT + LYS dressings, indicating enhanced wound healing and re-epithelialization. Furthermore, coccoid bacteria were predominantly observed in the untreated control groups and LYS-treated groups, suggesting that the OCT-based treatments were more effective in reducing bacterial surface colonization. Overall, the incorporation of OCT and LYS within the dressings demonstrated advantages over equivalent doses of their free forms, promoting tissue healing and managing infection without causing excessive tissue damage, thereby facilitating tissue remodeling and scar formation.

## 3 Conclusions

High anti-MRSA effects were observed for the synergetic combination of the OCT and LYS *in vitro* and *in vivo* using a murine excisional wound splinting model. It is possible to directly 3D-print antimicrobial wound dressings using fused deposition modeling and their antimicrobial action enhanced by electrostatically binding a lytic enzyme (LYS) encapsulated in polymer nanoparticles using PEI as polycation attached to the polymeric dressings. The surface properties of the resulting advanced PLA-based dressings can be modified with PEO to improve their wettability and hygroscopic character and to control the loaded antimicrobials release kinetics. Membrane polarization, cell lysis, and DNA release were bactericidal mechanisms identified for both antimicrobials in this study. Additionally, the combination of the anti-inflammatory effects of OCT and LYS loaded within PLA:PEO wound dressings demonstrated a superior therapeutic effectiveness *in vivo* compared to the effect of the same antimicrobials alone in their free form and when present individually in the dressings. Previously reported advanced dressings include antimicrobials and immunomodulators to promote re-epithelialization and fast wound healing; here, we identified synergism between OCT and LYS against MRSA together with an anti-inflammatory effect of OCT supporting regeneration over fibrosis *in vivo*. This advanced wound dressing can be manufactured using a conventional 3D printer and shows a multifunctional character both antimicrobial and anti-inflammatory thanks to the biological effects of the selected antimicrobials.

## 4 Materials and Methods

### 4.1 Materials

PLA (polylactic acid, MW 80 kDa), PEO (polyethylene oxide) (MW 100 kDa), chlorhexidine (>99.5%), polyethylenimine (branched, MW 25 kDa), and lysostaphin (from *Staphylococcus simulans*) were purchased from Sigma-Aldrich (St. Louis, MO, USA). Dichloromethane (DCM > 99%), fusidic acid (98%), and octenidine dihydrochloride (98%) were obtained from Thermo Fisher Scientific (Waltham, MA, USA). Tryptone soy broth (TSB) and tryptone soy agar (TSA) were obtained from Laboratorios Conda-Pronadisa S.A. (Madrid, Spain). Dulbecco’s modified Eagle medium (4.5 g/L) (DMEM) containing l-glutamine (2 mM), antibiotics (1% penicillin–streptomycin–amphotericin), and phosphate buffered saline (PBS) were obtained from Biowest (Cedex, France). Fetal bovine serum (FBS, 10% v/v) was used to supplement DMEM and was purchased from Gibco (Waltham, MA, USA).

### 4.2 Evaluation of the Thermal Stability and Bactericidal Effects of Selected Antimicrobial Drugs

We initially screened which antimicrobials could be suitable for the simultaneous fused deposition modeling (FDM) 3D printing of a polymer and an antimicrobial. Due to the high temperature (210–220 °C) reached during the thermal extrusion in the printer head, some antimicrobials could be degraded and their activity lost. Given the potentially low antimicrobial loading achieved when immersing an already printed dressing in an antimicrobial solution, we decided instead to include the antimicrobial within the polymer in the feed of the 3D printer. By doing so, all of the printed material would include the selected antimicrobial in its structure. Therefore, prior to the fabrication of the 3D printed antimicrobial-loaded wound dressings an *in vitro* preliminary study was conducted to determine the bactericidal effects of different commonly used topical antimicrobial compounds against MRSA, including two antiseptics chlorhexidine and octenidine dihydrochloride (OCT) and one antibiotic (fusidic acid) before and after a simulated thermal treatment mimicking the one occurring in the printer extruder. The three selected antimicrobials were treated at 220 °C for 5 min (conditions reached in the printer head during printing) in a Memmert BE300 oven (Memmert GmbH + Co., Germany). Their antimicrobial susceptibilities before and after heating were analyzed using the broth microdilution method on MRSA. To do so, MRSA (USA300, kindly donated by Cristina Prat-Aymerich, Ph.D., M.D., at IGTP, Badalona, Spain) suspensions at 10^5^ CFU/mL in TSB media containing serial concentrations of the corresponding antimicrobial were prepared before and after been heated up. After 24 h of incubation at 37 °C under stirring (150 rpm), cultures were diluted in PBS in 96-well microplates and subsequently spot-plated into TSA plates being the observed colonies counted after 24 h of incubation at 37 °C. All experiments were made in triplicate. Samples not treated with any antimicrobial were used as positive control. In parallel, the thermal stability of those antimicrobials was studied by thermogravimetric analysis (TGA) using a Mettler Toledo TGA/SDTA 851 equipment in air, from 35 to 600 °C with a heating rate of 10 °C/min.

### 4.3 Octenidine Dihydrochloride and Lysostaphin Synergy Evaluation

The *in vitro* antimicrobial evaluation of the potential synergy between OCT and LYS was determined by the checkerboard microdilution method.^64,65^ Individual minimum inhibitory concentrations (MICs) for both antimicrobials were initially calculated using the broth microdilution method following the same protocol mentioned above, but in this case, serial concentrations of the corresponding antimicrobial (OCT or LYS) were prepared. After 24 h of incubation at 37 °C under stirring (150 rpm), cultures were diluted in PBS in 96-well microplates and subsequently spot-plated into TSA plates being the observed colonies counted after 24 h of incubation at 37 °C. All experiments were made in triplicate. Samples not treated with any antimicrobial were used as positive control.

To evaluate their potential synergy, stock solutions containing the resulting individual MICs increased by 4-fold for both antimicrobials were prepared and diluted horizontally and vertically, respectively. We added 20 μL of the MRSA strain, achieving a final concentration of 10^5^ CFU/mL, to each well and used a bacteria-free mirror plate as the negative control. Subsequently, the plates were incubated at 37 °C for 24 h. Plates were centrifuged at 1500 rpm for 5 min and the supernatants were discarded. Two washes with PBS were performed before using the Blue Cell Viability Assay Kit (Abnova, Taiwan) to evaluate cellular viability in each well. Bacteria were incubated for 2 h in TSB with 10% of the reagent and subsequently the fluorescence emitted at 590 nm was quantified using a Varioskan LUX microplate reader (Thermo Scientific, Waltham, USA). Using the following equations, the fractional inhibitory concentration index (FICI) was calculated:

1 $$\mathrm{FICI}={\mathrm{FIC}}_{\mathrm{OCT}}+{\mathrm{FIC}}_{\mathrm{LYS}}$$

where

2 $${\mathrm{FIC}}_{\mathrm{OCT}}=\frac{{\mathrm{MIC}}_{\mathrm{OCT}}\mathrm{in combination with LYS}}{{\mathrm{MIC}}_{\mathrm{OCT}}}$$

3 $${\mathrm{FIC}}_{\mathrm{LYS}}=\frac{{\mathrm{MIC}}_{\mathrm{LYS}}\mathrm{in combination with OCT}}{{\mathrm{MIC}}_{\mathrm{LYS}}}$$

The interaction between both antimicrobials was identified as synergetic (FICI < 0.5), additive (0.5 ≤ FICI ≤ 1), indifferent (1 < FICI ≤ 4), or antagonist (FICI > 4) depending on the resulting values.

### 4.4 Synthesis of 3D-Printed Antimicrobial-Loaded Dressings

As mentioned before, separately prepared OCT-containing pellets were synthesized to load the printer feeder located in the head extruder. In brief, a solution of 10 g of PLA with 2% w/w OCT (PLA-OCT) were dissolved in 50 mL DCM at 700 rpm overnight. The solution was left to evaporate to reach a semisolid texture, which was extruded through a 20 mL syringe to obtain a 2 mm diameter filament, which was cut into approximately 5 mm long pieces to obtain pellets that were dried for 24 h at RT. Mixtures of PLA with PEO at different concentrations (3:1, 6:1, and 9:1) were also prepared and dissolved in 70 mL of DCM. PEO was introduced into the polymeric blends to increase the hydrophilicity of the resulting dressings in order to promote the absorption of wound exudates and to favor bacterial contact. In addition, 2% w/w OCT was also introduced into PLA:PEO (9:1) blends (named PLA:PEO-OCT) and corresponding pellets prepared following the same protocol described above. 2% w/w OCT loading in the printed dressings was chosen to inhibit bacterial growth while preserving eukaryotic cells viability.

The 3D printed dressings (2 mm × 2 mm × 0.5 mm) were designed using Paint 3D (Microsoft) and then imported into the PrusaSlicer open-source program. PLA, PLA-OCT, PLA:PEO and PLA:PEO-OCT dressings were printed in an Anycubic Chiron 3D printer (Shenzhen, China) at an extrusion temperature of 210–220 °C, having a nozzle diameter of 0.4 mm, using a print bed temperature of 50–60 °C and a printing speed of 3 mm/s, travel speed of 60 mm/s, and accuracy of 0.05–0.3 mm. The printer has incorporated a pellet extruder, which avoids the need of using a filament. The thickness of the dressings could be controlled, depending on the number of layers deposited. A mesh type pattern was used having a layer thickness of 0.25 mm, leaving an infill density of 90%.

Unlike OCT, it was not possible to incorporate LYS into the polymeric pellets used for 3D-printing due to its enzymatic nature because it would be completely denatured at the printing temperature (210–220 °C). Therefore, LYS was separately incorporated into the previously prepared OCT-loaded 3D printed dressings. LYS-loaded PLGA nanoparticles were separately prepared following our previously reported protocol.^26^ As we mentioned before, we used PLGA nanoparticles to encapsulate LYS to avoid its reported immunogenicity and fast degradation under physiological conditions in its free form. The LYS-loaded PLGA nanoparticles were electrostatically linked to the printed dressings by supramolecular interactions using PEI as a polycation. Printed dressings with a thickness of 0.25 mm and a diameter of 8 mm were immersed in 1 mL of PEI solution (1 mg/mL) for 15 min. Then, two washes were performed to remove nonbound PEI, and the resulting positively charged dressings were subsequently introduced in an aqueous dispersion containing LYS-loaded PLGA nanoparticles at a nanoparticle concentration of 2.5 or 5 mg/mL. After this process, the dressings were washed twice to remove weakly or nonattached nanoparticles. The final antimicrobial-loaded dressings were denoted as PLA:PEO + LYS and PLA:PEO-OCT + LYS.

### 4.5 Physicochemical Characterization of the 3D Printed Dressings

To evaluate the increase in the hydrophilic character of the PLA printed dressings after the introduction of PEO, we performed contact angle measurements. At room temperature, a 10 uL droplet of distilled water was deposited on the surface of the corresponding printed dressings for 1 min, followed by contact angle measurements utilizing Dataphysics OCA equipment (Dataphysics Instruments GmbH, Filderstadt, Germany). In addition, the presence of characteristic chemical bonds on the printed dressings was analyzed by using a Vertex 70 infrared spectrometer (Bruker, Bremen, Germany) with attenuated total reflection device (ATR Golden gate) (Specat Ltd., Orpington United Kingdon). For mechanical testing, the dressings were cut into 50 mm × 5 mm strips according to UNE-EN ISO 527-1:2012 (Plastics: Determination of tensile properties) norm. The mechanical properties of the dressings were assessed by a tensile test to determine the elastic modulus using an Instron Microtester 5548 (Instron, Norwood, USA) together with a laser video extensometer (at a speed of 1 mm/min with a load cell of 1 kN) at room temperature. Finally, the printed dressings were coated with Pt (∼10 nm) to analyze their morphological structure by scanning electron microscopy (SEM Inspect-F50; FEI Co. Hillsboro, OR, USA).

### 4.6 Antimicrobial Evaluation of the 3D-Printed Dressings

To evaluate the antimicrobial efficiency of the printed dressings, we followed the same broth dilution method described above but with slight modifications. A MRSA colony was picked, inoculated into 4 mL of TSB, and incubated at 37 °C under stirring (150 rpm) overnight. We diluted 1/100 a stationary growth phase culture of this MRSA suspension (∼10^9^ CFU/mL, OD_600nm_ = 1.4–1.6) to reach ≈10^5^ CFU/mL in a final volume of 20 mL. CFUs were counted after the bacteria were incubated at 37 °C under stirring for 24 h with the corresponding dressing. Subsequently, the serial microdilution method was used to evaluate the number of viable colonies. MICs and MBCs (minimum bactericidal concentrations) were determined. For the study of dressing durability, a dilution of 10^5^ CFU/mL was made and confronted with a dressing in a final volume of 20 mL and incubated for 48 h with agitation at 37 °C. After the first 24 and 48 h, plating was performed, and the number of colonies was counted. Subsequently, the same used dressing was washed with PBS to eliminate any bacteria that might remain adhered and was reapplied in a new bacterial medium having a fresh inoculum, and the process described above was repeated until the seventh day. Consequently, the same dressing faced three bacterial inoculums over a 1 week period.

### 4.7 Evaluation of the Mechanisms of Antimicrobial Action on MRSA

To evaluate the mechanism of bactericidal action of OCT and LYS on MRSA, bacterial cultures were incubated for 4 and 24 h at 37 °C under shaking (150 rpm) at the previously determined MIC and MBC concentrations. To study the influence of LYS and OCT on the cell membrane potential, a flow cytometer (Gallios flow cytometer, Beckman Coulter, USA) was used using the cellular pellets of the treated and nontreated bacteria (used as control) measuring mitochondrial membrane potentials. Samples were also centrifuged at 3500 rpm for 5 min, and the supernatant was collected for the analysis of nucleic acid release. The pellet was resuspended in 100 μL of PBS, and 5 μL of mitoStep (Immunostep, Salamanca, Spain) was added. Samples were incubated at 37 °C for 15 min. Subsequently, 400 μL of PBS and 0.5 μL of propidium iodide stain were added before being analyzed in a flow cytometer. For DNA extraction, 250 μL of 5 M NaCl was added to 500 μL of the previously collected bacterial supernatant, followed by centrifugation at 12 500 rpm for 15 min. The supernatant was treated with 500 μL of isopropanol and centrifuged again at 12 500 rpm for 15 min. The supernatant was discarded, and the pellet was washed with 500 μL of 70% EtOH, followed by centrifugation at 12 500 rpm for 15 min. The pellet was allowed to dry and was resuspended in 50 μL of H_2_O for further analysis. For the preparation of agarose gels, 2% agarose (Thermo Fisher Scientific, Waltham, MA, USA) was dissolved in 100 mL 1× TBE (Tris-borate EDTA) buffer and heated until complete dissolution. 2.5 μL of SYBR DNA dye (Thermo Fisher Scientific, Waltham, MA, USA) was added until the gel was formed. For sample preparation, 4–7 μL of extracted DNA was mixed with 1.5 μL of the dye and with 100 bp DNA ladder (BIORON GmbH, Römerberg, Germany) used as control. Electrophoresis was carried out with a PowerPac Universal Power Supply (Bio-Rad, Hercules, CA, USA) at a voltage of 80 V and displayed in the GelDoc go Imaging System (Bio-Rad, Hercules, CA, USA). To analyze the ability of OCT to degrade DNA, MRSA DNA was extracted and mixed with OCT at MIC and MBC concentrations for 2, 4, and 24 h. To analyze nucleic acid potential degradation, Qubit ssDNA and dsDNA high sensitivity assay kits (Thermo Fisher Scientific, Waltham, MA, USA) were used. In all experiments, the quality of the DNA was determined by measuring the 260/280 and 260/230 nm absorbance ratios with a Nanodrop 2000 spectophotometer (Thermo Fisher Scientific, Waltham, MA, USA).

### 4.8 Antimicrobial Release Kinetics

To study the amount of OCT released from the dressings over time, kinetics were evaluated in different solutions under sink conditions including Milli Q water with 2 wt % Tween-20, DMEM high glucose and in TSB. Different release media were used to corroborate that after heating, the OCT dihydrochloride became hydrophobic due to the loss of the dihydrochloride group. PLA, PLA-OCT, PLA:PEO, and PLA:PEO-OCT dressings were weighed, immersed in 20 mL of each of the mentioned solutions, and left to incubate at 37 °C on a shaker. Then, measurements were performed spectrophotometrically at 280 nm on the supernatants and at different times so that, subsequently, the concentration of the OCT was obtained by using a calibration curve separately implemented with known concentrations of this antiseptic.

### 4.9 Epithelial Cells Viability

Human epidermal keratinocytes (HaCaT; kindly donated by Dr. Pilar Martin-Duque), human dermal fibroblasts (Lonza, Basel Switzerland), and the murine macrophage cell line J774A.1 (ATCC TIB-67; LGC Standards, Spain) were used to determine the subcytotoxic doses of the free antimicrobials and those for the antimicrobial-loaded wound dressings. Cells were cultured in complete DMEM and incubated at 37 °C and 5% CO_2_ until confluence. The cytotoxicity of free OCT, free LYS, and the one of the exudates released from the dressings (PLA-OCT, PLA:PEO-OCT, and PLA:PEO-OCT-LYS) after 24, 48, and 72 h was evaluated in culture after dilution in complete medium. After 24 h, cell viability was evaluated by adding 10% Blue Cell Viability Assay Kit (Abnova, Taiwan) to the wells after incubation at 37 °C and 5% CO_2_ for 4 h. The fluorescence was then quantified, and the results were normalized with respect to untreated controls.

### 4.10 Anti-Inflammatory Drug Analysis

The murine macrophage cell line J774A.1 was seeded at a density of 60 000 cells/cm^2^ in 12-well plates in complete DMEM and incubated at 37 °C and 5% CO_2_ for 24 h. To induce inflammation, cells were treated with 100 ng/mL lipopolysaccharide (LPS) in serum-containing medium for 3 h. Subsequently, different concentrations of OCT were added, and the mixtures were incubated for 24 h. Then, 100 μL of the supernatant was collected from each well and 100 μL of Griess reagent was added to quantify the nitric oxide produced which is commonly associated with the inflammatory nitrosative stress. The plate was incubated at room temperature for 15 min. Finally, the absorbance was measured at 540 nm using a plate reader. NO production was calculated by interpolating the absorbance values of each sample with a sodium nitrite standard curve.

### 4.11 Quantitative Real-Time Reverse Transcriptase Polymerase Chain Reaction (qRT-PCR) analysis

To study the changes in the expression of pro-inflammatory genes, J774 cells were seeded at a density of 21 000 cells/cm^2^ in a 6-well plate. The induction of inflammation described above was repeated, and a post-treatment with OCT at its MIC (1 μg/mL) was performed. Cells were washed with PBS, and 500 μL of Trizol (ThermoFisher, Waltham, USA) was added to each well to lyse the cells before being transferred to 1.5 mL tubes. 100 μL of chloroform (Sigma-Aldrich, St. Louis, USA) was added to each tube and centrifuged at 12 000 rpm for 15 min at 4 °C to separate the phases. 200 μL of the upper phase was collected, and 0.5 μL of glycogen (ThermoFisher, Waltham, USA) was added. The samples were incubated for 10 min at RT. Subsequently, 250 μL of isopropanol (ThermoFisher, Waltham, USA) was added and centrifuged at 12 000 rpm for 10 min to precipitate the RNA and discard the supernatant. 500 μL of absolute ethanol (Sigma-Aldrich, St. Louis, USA) was added and centrifuged at 7500 rpm for 5 min to discard the supernatant and resuspend the pellet in 20 μL of H_2_O. RNA quantification was performed at 260 nm using a NanoDrop 2000 instrument (Thermo Fisher Scientific, Waltham, USA). For RNA retrotranscription to cDNA, the PrimeScript RT Master Mix kit (Takara Bio, Shiga, Japan) was used with 500 ng of the extracted RNA. Relative quantification of the obtained cDNA after RT-PCR was performed by quantitative PCR (qPCR) using Premix Ex Taq DNA polymerase (Takara Bio, Shiga, Japan) as detection method, and the different studied genes were amplified using specific primers (Table 1). The fluorescence emitted during the PCR reaction was detected with a QuantStudio 5 System instrument (Thermo Fisher Scientific). For data analysis the mathematical method delta-delta-Ct (2-ΔΔΔCt) was used for the relative quantification of our interest genes.^66^ Study genes were normalized with the endogenous control Actb and expressed as a percentage using LPS as reference (100%).

**Table 1:** Mouse Gene Primers Used in qPCR for the Study of Pro-Inflammatory Genes Acquired in Integrated DNA Technologies (Newark, NJ, USA)

mouse genes	target probes
Actb	Mm.PT.39a.22214843.g
IL-1β	Mm.PT.58.41616450
IL-6	Mm.PT.58.10005566
TNF-α	Mm.PT.58.29509614
Nos2	Mm.PT.58.43705194

### 4.12 *In Vivo* Study

*In vivo* experiments were conducted in accordance with the Spanish and European regulation (Royal Decree 53/2013 and EU Directive 2010/63) on the protection of animals used for scientific purposes and approved by the University of Zaragoza ethical review committee (PI20/24) designated as Animal Welfare Body. This study included male and female SKH1 hairless mice aged between 7 and 10 weeks (Charles River Laboratories, Wilmington, DE, USA). All mice were maintained in BSL2 laboratory and housed in individually ventilated cages with unlimited access to food and water. A murine excisional wound splinting model was developed to analyze the process of wound healing after wounding and infection. Silicone splint rings were used to prevent the natural wound contraction of the murine skin. A total of 28 animals were divided into seven experimental groups (*N* = 4):

I. Infected mice without any treatment (control group)
II. Infected mice treated with free OCT (25 μL at a concentration of 25 mg/L)
III. Infected mice treated with PLA:PEO-OCT dressings (8 mm in diameter containing 0.2 mg of OCT)
IV. Infected mice treated with free LYS (25 μL at a concentration of 0.2 mg/L)
V. Infected mice treated with PLA:PEO + LYS
VI. Infected mice treated with a combination of free OCT and LYS (25 μL containing 6.25 μg/L LYS and 0.125 mg/L OCT in PBS)
VII. Infected mice treated with PLA:PEO-OCT + LYS (8 mm in diameter containing 0.2 mg OCT and 0.8 μg)

The weight of the animals was monitored before surgery and daily throughout the study to detect any potential weight loss. Mice were anesthetized with 4% isoflurane for induction and maintained under 2% with an oxygen flow of 1L/min during the surgical procedures. Animals were treated immediately before surgery with a nonsteroidal anti-inflammatory analgesic drug (Meloxicam) with a dose of 5 mg/kg body weight subcutaneously, followed by every 24 h for 72 h postsurgery and infection (PSI).

The excisional wound splinting model was conducted as previously established in our group.^67^ Briefly, the animals’ skin was disinfected with rubbing alcohol, and two circular incisions were made in the scapular area using a sterile biopsy punch (⌀ = 8 mm; Eickemeyer Veterinary Equipment Ltd., Stratford, Canada). Silicone wound splints (14 mm OD × 10 mm ID × 0.5 mm thick; Grace Bio-Labs, Bend, USA) were sutured around the wounds using a polyamide suture (Dafilon 4/0; Braun, Germany). The wounds were then inoculated with a dispersion of MRSA USA300 (25 μL, ≈10^4^ CFU/mL in PBS) to induce infection, followed by the administration of the different treatments according to the corresponding experimental groups. This bacterial load was selected after a preliminary animal test (results not shown) to guarantee infection without producing sepsis or septic shock in the animals. Finally, the wounds were covered with sterile adhesive plasters (Hartmann, Heidenheim, Germany) and Tegaderm (3M, Saint Paul, MN, US). All treatments were reapplied at 2 and 4 days of PSI mimicking conventional clinical procedures where bandage replacements are intended to keep the wound clean and to absorb exudates.

Infection progression, wound healing, body weight changes, and animal welfare were monitored daily until the end of the study. Wounds were photographed daily to document healing and the infection progression. Wound infection presence was assessed by collecting microbiological samples from the wounds at 2, 4, and 7 days of PSI with swabs (Deltalab, Barcelona, Spain). The samples were then cultured in Brillance MRSA2 chromogenic agar plates (Thermo Fisher Scientific, Waltham, MA, USA), specifically designed for MRSA isolation. A streak plate method was used to perform a semiquantitative analysis to estimate the bacterial load in the animals’ wounds. The mechanical dilution of the microbial sample over the surface of the solid agar was performed by using an inoculating loop. This dilution takes place through sequential streaking across different quadrants of the plate, which reduces cell density, and then individual bacterial cells are spatially separated and can grow into discrete colonies. Each visible colony theoretically originates from a single colony-forming unit (CFU): a viable bacterial cell or a cluster of cells. Based on the extent of bacterial growth on the plate quantified, the bacterial load was classified as follows: (0) no growth and from (1) to (4) for increasing levels of bacterial presence (1 minor, 2 moderate, 3 extensive, and 4 massive). On day 7 PSI, mice were euthanized via CO_2_ inhalation, and the wounds along with surrounding tissues were collected, fixed in PBS with paraformaldehyde (4% w/v) (Alfa Aesar, Heysham, U.K.) for 24 h, and embedded in paraffin. Lastly, histological sections (5 μm thick) were stained with hematoxylin and eosin (HE) and Gram stain before pathological analysis.

### 4.13 Statistical Analysis

All values are expressed as the mean ± standard deviation. Differences between two groups were compared by the Student’s *t* test, and differences between three or more groups were analyzed by one-way or two-way ANOVA tests using GraphPadPrism software (version 7.00; GraphPad software). Differences were considered significant with an adjusted *p*-value of <0.05.

## Supplementary Material



## Data Availability

Data will be made available upon request.

## Notes

Figure S1 showing SEM images, frequency distribution, and drug release profile and Table S1 listing histopathological results (PDF)

## References

[1] Murray C. J. L., Ikuta K. S., Sharara F., Swetschinski L., Robles Aguilar G., Gray A., Han C., Bisignano C., Rao P., Wool E., Johnson S. C., Browne A. J., Chipeta M. G., Fell F., Hackett S., Haines-Woodhouse G., Kashef Hamadani B. H., Kumaran E. A. P., McManigal B., Achalapong S., Agarwal R., Akech S., Albertson S., Amuasi J., Andrews J., Aravkin A., Ashley E., Babin F.-X., Bailey F., Baker S., Basnyat B., Bekker A., Bender R., Berkley J. A., Bethou A., Bielicki J., Boonkasidecha S., Bukosia J., Carvalheiro C., Castañeda-Orjuela C., Chansamouth V., Chaurasia S., Chiurchiù S., Chowdhury F., Clotaire Donatien R., Cook A. J., Cooper B., Cressey T. R., Criollo-Mora E., Cunningham M., Darboe S., Day N. P. J., De Luca M., Dokova K., Dramowski A., Dunachie S. J., Duong Bich T., Eckmanns T., Eibach D., Emami A., Feasey N., Fisher-Pearson N., Forrest K., Garcia C., Garrett D., Gastmeier P., Giref A. Z., Greer R. C., Gupta V., Haller S., Haselbeck A., Hay S. I., Holm M., Hopkins S., Hsia Y., Iregbu K. C., Jacobs J., Jarovsky D., Javanmardi F., Jenney A. W. J., Khorana M., Khusuwan S., Kissoon N., Kobeissi E., Kostyanev T., Krapp F., Krumkamp R., Kumar A., Kyu H. H., Lim C., Lim K., Limmathurotsakul D., Loftus M. J., Lunn M., Ma J., Manoharan A., Marks F., May J., Mayxay M., Mturi N., Munera-Huertas T., Musicha P., Musila L. A., Mussi-Pinhata M. M., Naidu R. N., Nakamura T., Nanavati R., Nangia S., Newton P., Ngoun C., Novotney A., Nwakanma D., Obiero C. W., Ochoa T. J., Olivas-Martinez A., Olliaro P., Ooko E., Ortiz-Brizuela E., Ounchanum P., Pak G. D., Paredes J. L., Peleg A. Y., Perrone C., Phe T., Phommasone K., Plakkal N., Ponce-de-Leon A., Raad M., Ramdin T., Rattanavong S., Riddell A., Roberts T., Robotham J. V., Roca A., Rosenthal V. D., Rudd K. E., Russell N., Sader H. S., Saengchan W., Schnall J., Scott J. A. G., Seekaew S., Sharland M., Shivamallappa M., Sifuentes-Osornio J., Simpson A. J., Steenkeste N., Stewardson A. J., Stoeva T., Tasak N., Thaiprakong A., Thwaites G., Tigoi C., Turner C., Turner P., van Doorn H. R., Velaphi S., Vongpradith A., Vongsouvath M., Vu H., Walsh T., Walson J. L., Waner S., Wangrangsimakul T., Wannapinij P., Wozniak T., Young Sharma T. E. M.
W., Yu K. C., Zheng P., Sartorius B., Lopez A. D., Stergachis A., Moore C., Dolecek C., Naghavi M. (2022). Global Burden of Bacterial
Antimicrobial Resistance in 2019: A Systematic Analysis. Lancet.

[2] Scharn C. R., Tickler I. A., Tenover F. C., Goering R. V. (2022). Characterization
of SCC *Mec* Instability in Methicillin-Resistant Staphylococcus
Aureus Affecting Adjacent Chromosomal Regions, Including the Gene
for Staphylococcal Protein A (*Spa*). Antimicrob. Agents Chemother..

[3] Merk, H. ; Diaz Högberg, L. ; Plachouras, D. ; Suetens, C. ; Monnet, D. L. Assessing the Health Burden of Infections with Antibiotic-Resistant Bacteria in the EU/EEA, 2016–2020. ECDC: Stockholm, 2020; 10.2900/73460.

[4] Piewngam P., Otto M. (2024). Staphylococcus Aureus Colonisation and Strategies for Decolonisation. Lancet Microbe.

[5] Bhattacharya S. (2016). Surgical Site
Infection by Methicillin Resistant Staphylococcus Aureus– on
Decline?. J. Clin Diagn Res..

[6] Rutz J., Naendrup J.-H., Bruns C., Classen A. Y., Salmanton-García J., Seifert H., Sprute R., Stemler J., Walker S. V., Cornely O. A., Liss B. J., Mellinghoff S. C., Ankert J., Bernard L., Bataille C., Couvé-Deacon E., Ferrer M. F., Fortún J., Galar A., Guimard T., Horcajada J. P., Mollar J., Muñoz P., Pletz M. W., Serracino-Inglott F., Soriano A., Vilz T. O. (2024). Individual
and Institutional Predisposing Factors of MRSA Surgical Site Infection
and Outcomes—a Retrospective Case-Control-Study in 14 European
High-Volume Surgical Centres. JAC Antimicrob.
Resist..

[7] Fukuda H., Sato D., Iwamoto T., Yamada K., Matsushita K. (2020). Healthcare
Resources Attributable to Methicillin-Resistant Staphylococcus Aureus
Orthopedic Surgical Site Infections. Sci. Rep.

[8] Allareddy V., Das A., Lee M. K., Nalliah R. P., Rampa S., Allareddy V., Rotta A. T. (2015). Prevalence, Predictors, and Outcomes of Methicillin-Resistant
Staphylococcus Aureus Infections in Patients Undergoing Major Surgical
Procedures in the United States: A Population-Based Study. Am. J. Surg.

[9] Macdonald K. E., Boeckh S., Stacey H. J., Jones J. D. (2021). The Microbiology
of Diabetic Foot Infections: A Meta-Analysis. BMC Infect Dis.

[10] Wolcott R. D., Hanson J. D., Rees E. J., Koenig L. D., Phillips C. D., Wolcott R. A., Cox S. B., White J. S. (2016). Analysis
of the
Chronic Wound Microbiota of 2,963 Patients by 16S RDNA Pyrosequencing. Wound Repair Regen.

[11] Zhou S., Hu X., Wang Y., Fei W., Sheng Y., Que H. (2024). The Global
Prevalence of Methicillin-Resistant Staphylococcus Aureus in Patients
with Diabetic Foot Ulcers: A Systematic Review and Meta-Analysis. Diabetes Metab Syndr Obes.

[12] Reich-Schupke S., Warneke K., Altmeyer P., Stücker M. (2010). Eradication
of MRSA in Chronic Wounds of Outpatients with Leg Ulcers Is Accelerated
by Antiseptic Washes – Results of a Pilot Study. Int. J. Hyg Environ. Health.

[13] Uçkay I., Kressmann B., Malacarne S., Toumanova A., Jaafar J., Lew D., Lipsky B. A. (2018). A Randomized, Controlled
Study to Investigate the Efficacy and Safety of a Topical Gentamicin-Collagen
Sponge in Combination with Systemic Antibiotic Therapy in Diabetic
Patients with a Moderate or Severe Foot Ulcer Infection. BMC Infect. Dis..

[14] Vas P., Rayman G., Dhatariya K., Driver V., Hartemann A., Londahl M., Piaggesi A., Apelqvist J., Attinger C., Game F. (2020). Effectiveness of Interventions to
Enhance Healing of Chronic Foot Ulcers in Diabetes: A Systematic Review. Diabetes Metab. Res. Rev..

[15] Weigelt M. A., McNamara S. A., Sanchez D., Hirt P. A., Kirsner R. S. (2021). Evidence-Based
Review of Antibiofilm Agents for Wound Care. Adv. Wound Care (New Rochelle).

[16] Atkin L., Bućko Z., Montero E. C., Cutting K., Moffatt C., Probst A., Romanelli M., Schultz G. S., Tettelbach W. (2019). Implementing
TIMERS: The Race against Hard-to-Heal Wounds. J. Wound Care.

[17] Falcone M., De Angelis B., Pea F., Scalise A., Stefani S., Tasinato R., Zanetti O., Dalla Paola L. (2021). Challenges
in the Management of Chronic Wound Infections. J. Glob Antimicrob Resist.

[18] Duarte-Mata D. I., Salinas-Carmona M. C. (2023). Antimicrobial Peptides′ Immune
Modulation Role
in Intracellular Bacterial Infection. Front.
Immunol..

[19] Wang G., Li X., Wang Z. (2016). APD3: The Antimicrobial Peptide Database as a Tool
for Research and Education. Nucleic Acids Res..

[20] Malanovic N., Lohner K. (2016). Antimicrobial Peptides Targeting Gram-Positive Bacteria. Pharmaceuticals.

[21] Zhao H., Eszterhas S., Furlon J., Cheng H., Griswold K. E. (2021). Electrostatic-Mediated
Affinity Tuning of Lysostaphin Accelerates Bacterial Lysis Kinetics
and Enhances In Vivo Efficacy. Antimicrob. Agents
Chemother..

[22] Zhao H., Brooks S. A., Eszterhas S., Heim S., Li L., Xiong Y. Q., Fang Y., Kirsch J. R., Verma D., Bailey-Kellogg C., Griswold K. E. (2020). Globally Deimmunized Lysostaphin
Evades Human Immune Surveillance and Enables Highly Efficacious Repeat
Dosing. Sci. Adv..

[23] Desbois A. P., Gemmell C. G., Coote P. J. (2010). In Vivo
Efficacy of the Antimicrobial
Peptide Ranalexin in Combination with the Endopeptidase Lysostaphin
against Wound and Systemic Meticillin-Resistant Staphylococcus Aureus
(MRSA) Infections. Int. J. Antimicrob. Agents.

[24] Mohamed M. F., Seleem M. N. (2014). Efficacy of Short Novel Antimicrobial and Anti-Inflammatory
Peptides in a Mouse Model of Methicillin-Resistant Staphylococcus
Aureus (MRSA) Skin Infection. Drug Des. Dev.
Ther.

[25] Yang X., Xie B., Peng H., Shi G., Sreenivas B., Guo J., Wang C., He Y. (2021). Eradicating Intracellular MRSA via
Targeted Delivery of Lysostaphin and Vancomycin with Mannose-Modified
Exosomes. J. Controlled Release.

[26] Landa G., Aguerri L., Irusta S., Mendoza G., Arruebo M. (2024). PLGA Nanoparticle-Encapsulated
Lysostaphin for the Treatment of Staphylococcus Aureus Infections. Int. J. Biol. Macromol..

[27] Li C., Xiong Y., Fu Z., Ji Y., Yan J., Kong Y., Peng Y., Ru Z., Huang Y., Li Y., Yang Y., He L., Tang J., Wang Y., Yang X. (2024). The Direct Binding
of Bioactive Peptide Andersonin-W1 to TLR4 Expedites
the Healing of Diabetic Skin Wounds. Cell Mol.
Biol. Lett..

[28] Müller G., Kramer A. (2008). Biocompatibility Index of Antiseptic Agents by Parallel
Assessment of Antimicrobial Activity and Cellular Cytotoxicity. J. Antimicrob. Chemother..

[29] Krishna B. V. S., Gibb A. P. (2010). Use of Octenidine
Dihydrochloride in Meticillin-Resistant
Staphylococcus Aureus Decolonisation Regimens: A Literature Review. J. Hosp Infect.

[30] Cassiano A. F. B., Coaguila-Llerena H., Santos C. S., da Silva L. R., Nogueira L. F. B., Ciancaglini P., Faria G. (2022). The Effect of Octenidine
on Proliferation, Migration, and Osteogenic Differentiation of Human
Dental Pulp and Apical Papilla Stem Cells. J.
Endod..

[31] Rzycki M., Drabik D., Szostak-Paluch K., Hanus-Lorenz B., Kraszewski S. (2021). Unraveling the Mechanism of Octenidine
and Chlorhexidine
on Membranes: Does Electrostatics Matter?. Biophys.
J..

[32] Malanovic N., Buttress J. A., Vejzovic D., Ön A., Piller P., Kolb D., Lohner K., Strahl H. (2022). Disruption
of the Cytoplasmic Membrane Structure and Barrier Function Underlies
the Potent Antiseptic Activity of Octenidine in Gram-Positive Bacteria. Appl. Environ. Microbiol..

[33] Al-Doori Z., Goroncy-Bermes P., Gemmell C. G., Morrison D. (2007). Low-Level Exposure
of MRSA to Octenidine Dihydrochloride Does Not Select for Resistance. J. Antimicrob. Chemother..

[34] Stahl J., Braun M., Siebert J., Kietzmann M. (2010). The Effect
of a Combination of 0.1% Octenidine Dihydrochloride and 2% 2-Phenoxyethanol
(Octenisept®) on Wound Healing in Pigs in Vivo and Its in Vitro
Percutaneous Permeation through Intact and Barrier Disrupted Porcine
Skin. Int. Wound J..

[35] Seiser S., Janker L., Zila N., Mildner M., Rakita A., Matiasek J., Bileck A., Gerner C., Paulitschke V., Elbe-Bürger A. (2021). Octenidine-Based
Hydrogel Shows Anti-Inflammatory and
Protease-Inhibitory Capacities in Wounded Human Skin. Sci. Rep.

[36] Huang J., Fan Q., Guo M., Wu M., Wu S., Shen S., Wang X., Wang H. (2021). Octenidine Dihydrochloride
Treatment
of a Meticillin-Resistant Staphylococcus Aureus Biofilm-Infected Mouse
Wound. J. Wound Care.

[37] Hämmerle G., Strohal R. (2016). Efficacy and Cost-Effectiveness
of Octenidine Wound
Gel in the Treatment of Chronic Venous Leg Ulcers in Comparison to
Modern Wound Dressings. Int. Wound J..

[38] Kusuma C., Jadanova A., Chanturiya T., Kokai-Kun J. F. (2007). Lysostaphin-Resistant
Variants of *Staphylococcus Aureus* Demonstrate Reduced
Fitness In Vitro and In Vivo. Antimicrob. Agents
Chemother..

[39] Kwiatkowski P., Łopusiewicz Ł., Kostek M., Drozłowska E., Pruss A., Wojciuk B., Sienkiewicz M., Zielińska-Bliźniewska H., Dołęgowska B. (2020). The Antibacterial
Activity of Lavender Essential Oil Alone and In Combination with Octenidine
Dihydrochloride against MRSA Strains. Molecules.

[40] Hardy K., Sunnucks K., Gil H., Shabir S., Trampari E., Hawkey P., Webber M. (2018). Increased
Usage of Antiseptics Is
Associated with Reduced Susceptibility in Clinical Isolates of *Staphylococcus Aureus*. mBio.

[41] Sousa F. F. O., Nojosa J. S., Alencar C. A. A., Alcantara A. P. M. P., Araújo R. S., Yamauti M., Rodrigues L. K. A. (2021). Design
and Characterization of Digluconate and Diacetate Chlorhexidine Loaded-PLGA
Microparticles for Dental Applications. J. Drug
Deliv Sci. Technol..

[42] Marian E., Tita B., Duteanu N., Vicas L., Ciocan S., Jurca T., Antal L., Tica O., Mureşan M., Pallag A., Micle O. (2020). Antimicrobial Activity
of Fusidic
Acid Inclusion Complexes. Int. J. Infect Dis.

[43] Royal Society of Chemistry. Chemspider.com. Octenidine dihydrochloride, CSID 46370. https://www.chemspider.com/Chemical-Structure.46370.Html (accessed Mar 10, 2025).

[44] Siriprom W., Sangwaranatee N., Herman, Chantarasunthon K., Teanchai K., Chamchoi N. (2018). Characterization
and Analyzation of the Poly (L-Lactic Acid) (PLA) Films. Mater. Today Proc..

[45] Zhang H., Shao C., Kong W., Wang Y., Cao W., Liu C., Shen C. (2017). Memory Effect
on the Crystallization Behavior of Poly­(Lactic
Acid) Probed by Infrared Spectroscopy. Eur.
Polym. J..

[46] Mondragón-Herrera L. I., Vargas-Coronado R. F., Carrillo-Escalante H., Cauich-Rodríguez J. V., Hernández-Sánchez F., Velasco-Santos C., Avilés F. (2024). Mechanical, Thermal, and Physicochemical Properties
of Filaments of Poly (Lactic Acid), Polyhydroxyalkanoates and Their
Blend for Additive Manufacturing. Polymers (Basel).

[47] Vrandečić N. S., Erceg M., Jakić M., Klarić I. (2010). Kinetic Analysis
of Thermal Degradation of Poly­(Ethylene Glycol) and Poly­(Ethylene
Oxide)­s of Different Molecular Weight. Thermochim.
Acta.

[48] Singla P., Mehta R., Berek D., Upadhyay S. N. (2012). Microwave
Assisted
Synthesis of Poly­(Lactic Acid) and Its Characterization Using Size
Exclusion Chromatography. J. Macromol. Sci.
A.

[49] Maggi L., Segale L., Torre M. L., Ochoa
Machiste E., Conte U. (2002). Dissolution Behaviour of Hydrophilic
Matrix Tablets Containing Two
Different Polyethylene Oxides (PEOs) for the Controlled Release of
a Water-Soluble Drug. Dimensionality Study. Biomaterials.

[50] Agaliotis E. M., Ake-Concha B. D., May-Pat A., Morales-Arias J. P., Bernal C., Valadez-Gonzalez A., Herrera-Franco P. J., Proust G., Koh-Dzul J. F., Carrillo J. G., Flores-Johnson E. A. (2022). Tensile
Behavior of 3D Printed Polylactic Acid (PLA) Based Composites Reinforced
with Natural Fiber. Polymers (Basel).

[51] Maikranz E., Spengler C., Thewes N., Thewes A., Nolle F., Jung P., Bischoff M., Santen L., Jacobs K. (2020). Different
Binding Mechanisms of Staphylococcus Aureus to Hydrophobic and Hydrophilic
Surfaces. Nanoscale.

[52] Fang Y., Kirsch J. R., Li L., Brooks S. A., Heim S., Tan C., Eszterhas S., Cheng H. D., Zhao H., Xiong Y. Q., Griswold K. E. (2021). Deimmunized
Lysostaphin Synergizes with Small-Molecule
Chemotherapies and Resensitizes Methicillin-Resistant *Staphylococcus
Aureus* to β-Lactam Antibiotics. Antimicrob. Agents Chemother..

[53] Desbois A. P., Coote P. J. (2011). Bactericidal Synergy of Lysostaphin in Combination
with Antimicrobial Peptides. Eur. J. Clin Microbiol
Infect Dis.

[54] Tang Y. W., Hon P. Y., Tan J., Poh B. F., Ang B., Chow A. (2024). Octenidine Exposure Was Not Associated with Reduced
Octenidine Susceptibility
of Meticillin-Resistant Staphylococcus Aureus in an Extended-Care
Facility in Singapore. J. Hosp Infect.

[55] Wojciechowski K., Klodzinska E. (2015). Zeta Potential
Study of Biodegradable Antimicrobial
Polymers. Colloids Surf. A Physicochem Eng.
Asp.

[56] Barick P., Prasad Saha B., Mitra R., Joshi S. V. (2015). Effect of Concentration
and Molecular Weight of Polyethylenimine on Zeta Potential, Isoelectric
Point of Nanocrystalline Silicon Carbide in Aqueous and Ethanol Medium. Ceram. Int..

[57] Schmidt J., Zyba V., Jung K., Rinke S., Haak R., Mausberg R. F., Ziebolz D. (2016). Cytotoxic
Effects of Octenidine Mouth
Rinse on Human Fibroblasts and Epithelial Cells – an *in Vitro* Study. Drug Chem. Toxicol.

[58] Han S., Gao H., Chen S., Wang Q., Li X., Du L. J., Li J., Luo Y. Y., Li J. X., Zhao L. C., Feng J., Yang S. (2019). Procyanidin A1 Alleviates Inflammatory Response Induced by LPS through
NF-KB, MAPK, and Nrf2/HO-1 Pathways in RAW264.7 Cells. Sci. Rep.

[59] Lima T. S. (2023). Beyond
an Inflammatory Mediator: Interleukin-1 in Neurophysiology. Exp Physiol.

[60] Lee C. W., Kim S. C., Kwak T. W., Lee J. R., Jo M. J., Ahn Y. T., Kim J. M., An W. G. (2012). Anti-Inflammatory
Effects of Bangpungtongsung-San, a Traditional Herbal Prescription. Evidence-Based Complementary Altern. Med..

[61] Johnson C. T., Wroe J. A., Agarwal R., Martin K. E., Guldberg R. E., Donlan R. M., Westblade L. F., García A. J. (2018). Hydrogel
Delivery of Lysostaphin Eliminates Orthopedic Implant Infection by *Staphylococcus aureus* and Supports Fracture Healing. Proc. Natl. Acad. Sci. U. S. A..

[62] Craft K. M., Nguyen J. M., Berg L. J., Townsend S. D. (2019). Methicillin-Resistant *Staphylococcus Aureus* (MRSA): Antibiotic-Resistance and
the Biofilm Phenotype. Medchemcomm.

[63] Lee C. K., de Anda J., Baker A. E., Bennett R. R., Luo Y., Lee E. Y., Keefe J. A., Helali J. S., Ma J., Zhao K., Golestanian R., O’Toole G. A., Wong G. C. L. (2018). Multigenerational Memory and Adaptive
Adhesion in Early
Bacterial Biofilm Communities. Proc. Natl. Acad.
Sci. U. S. A..

[64] European
Committee for Antimicrobial Susceptibility Testing (EUCAST) of the
European Society of Clinical Microbiology and Infectious Diseases
(ESCMID). (2000). Terminology Relating
to Methods for the Determination of Susceptibility of Bacteria to
Antimicrobial Agents. Clin Microbiol Infect.

[65] Odds F. C. (2003). Synergy,
Antagonism, and What the Chequerboard Puts between Them. J. Antimicrob. Chemother..

[66] Pfaffl M. W. (2001). A New Mathematical
Model for Relative Quantification in Real-Time RT-PCR. Nucleic Acids Res..

[67] Landa G., Miranda-Calderon L. G., Gomez A., Perez M., Sebastian V., Arruebo M., Lamarche I., Tewes F., Irusta S., Mendoza G. (2023). Real-Time in Vivo Monitoring of the Antimicrobial Action
of Combination Therapies in the Management of Infected Topical Wounds. Int. J. Pharm..

