# Supplementary material for: Multifunctional 3D-Printed Wound Dressings Containing a Combination of Synergistic Antimicrobials in the Management of MRSA Infected Topical Wounds
Source: ACS Appl Mater Interfaces. 2025 Aug 18;17(34):47951–68. doi: 10.1021/acsami.5c08968 (PMC12400267; doi:10.1021/acsami.5c08968)
Supplement: Supplementary file 1 [file am5c08968_si_001.pdf]

Supporting Information

Multifunctional 3D-printed wound dressings containing a combination of synergistic antimicrobials in the management of MRSA infected topical wounds

Iman Mattar[a,b,c], *Guillermo Landa*[a,b,c*], *Marina Frutos-Lizano*[b,c], *Natalia Izquierdo*[b,c], *Elena Tapia*[d], *Marta Perez*[e,f], *Lluis Lujan*[f,g], *Silvia Irusta*[a,b], *Gracia Mendoza*[c,h], *Manuel Arruebo*[a,b,c,*]

[a]Instituto de Nanociencia y Materiales de Aragón (INMA), CSIC-Universidad de Zaragoza, 50009 Zaragoza, Spain.

[b]Department of Chemical and Environmental Engineering. University of Zaragoza, Campus Río Ebro-Edificio I+D, C/ Poeta Mariano Esquillor S/N, 50018 Zaragoza, Spain.

[c]Aragon Health Research Institute (IIS Aragon), 50009 Zaragoza, Spain.[d]Animal Unit, University of Zaragoza, 50009, Zaragoza, Spain.

[e]Department of Anatomy, Embriology and Animal Genetics, University of Zaragoza, 177 Miguel Servet Street, 50013, Zaragoza, Spain.

[f]Instituto Universitario de Investigación Mixto Agroalimentario de Aragón (IA2), University of Zaragoza, 50013 Zaragoza, Spain.

[g]Department of Animal Pathology, University of Zaragoza, 177 Miguel Servet Street, 50013, Zaragoza, Spain.

[h]Department of Pharmacology and Physiology, Forensic and Legal Medicine, Veterinary Faculty, University of Zaragoza, 177 Miguel Servet Street, 50013 Zaragoza, Spain.

Corresponding authors: M.A. *E-mail address*: arruebom@unizar.es and G.L. guillermolanda94@gmail.com

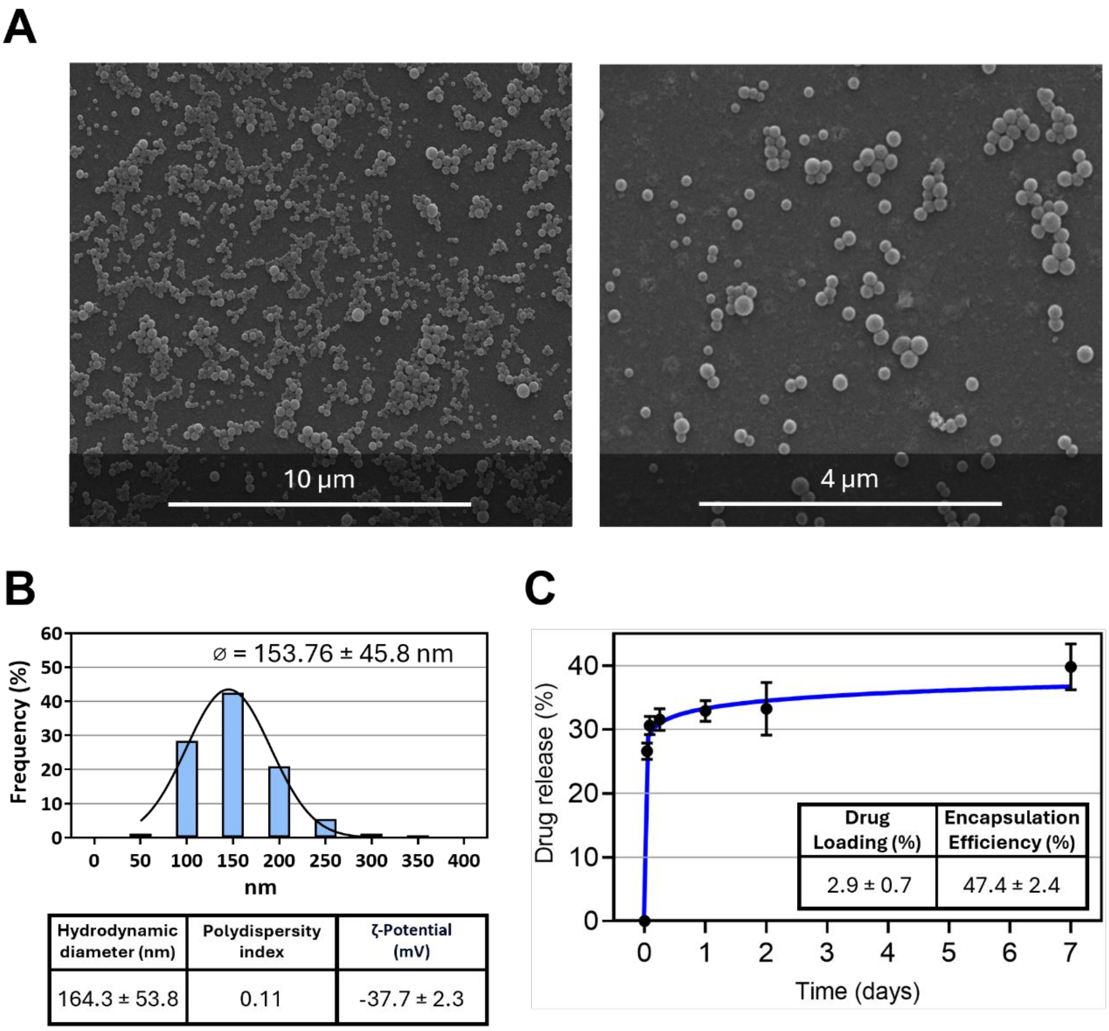

Figure S1. Synthesis and characterization of LYS-loaded PLGA nanoparticles (PLGA@LYS NPs). (A) SEM images of PLGA@LYS NPs (B) Frequency distribution of PLGA@LYS NPs diameters retrieved from the SEM images. Table showing the results of hydrodynamic diameters measured by dynamic light scattering and zeta potential measurements at pH 7. (C) Drug release profile of LYS from PLGA@LYS NPs, including numerical fitting using the Peppas–Sahlin model. The inset table presents the drug loading and encapsulation efficiency percentages.

Experimental Group	Tissue											
	Epidermis				Dermis		Subcutaneous					Bacterial presence
	Bacteria	Hyperplasia	Ulceration	Crust	Inflammation	Cell types	Fibroplasia	Panniculitis	Cell types	Mononuclear inflammation	Necrosis	Gram staining
**CTRL**	+	++	++	+	++	F	++	++	NFM	++	+	+
**OCT**	+	+	+	+	+	FN	++	+	NFM	-	+	+
**PLA:PEO-OCT**	-	+	-	+	-	-	+	+	FN	+	-	-
**LYS**	+++	+	++	+	++	FN	+	+++	FN	++	+++	+++
**PLA:PEO+LYS**	+	-	+	+	+	FN	+	+	FN	+	++	+
**OCT+LYS**	+	++	+	+	++	FN	++	++	FN	+	+	+
**PLA:PEO-OCT + LYS**	-	-	-	-	-/+	FN	+	+	FN	+	-	-

Table S1. Histopathological analysis of infected topical wounds. Evaluation was performed using tissue samples stained with hematoxylin-eosin and Gram stain, obtained from four different animals per experimental group. The analysis was conducted in a single-blind manner. Each parameter was scored on a semi-quantitative scale ranging from absence (−) to massive presence (++++). The types of inflammatory cells are indicated as follows: N, neutrophils; F, fibroblasts; M, macrophages; and L, lymphocytes.
